# Uncovering *Wolbachia* Diversity upon Artificial Host Transfer

**DOI:** 10.1371/journal.pone.0082402

**Published:** 2013-12-20

**Authors:** Daniela I. Schneider, Markus Riegler, Wolfgang Arthofer, Hervé Merçot, Christian Stauffer, Wolfgang J. Miller

**Affiliations:** 1 Laboratories of Genome Dynamics, Department of Cell- and Developmental Biology, Medical University of Vienna, Vienna, Austria; 2 Hawkesbury Institute for the Environment, University of Western Sydney, Penrith, Australia; 3 Molecular Ecology Group, Institute of Ecology, University of Innsbruck, Innsbruck, Austria; 4 UMR 7138, CNRS-Université Pierre & Marie Curie, Paris, France; 5 Institute of Forest Entomology, Forest Pathology and Forest Protection, Department of Forest & Soil Sciences, Boku, University of Natural Resources and Life Sciences, Vienna, Austria; University of Poitiers, France

## Abstract

The common endosymbiotic *Wolbachia* bacteria influence arthropod hosts in multiple ways. They are mostly recognized for their manipulations of host reproduction, yet, more recent studies demonstrate that *Wolbachia* also impact host behavior, metabolic pathways and immunity. Besides their biological and evolutionary roles, *Wolbachia* are new potential biological control agents for pest and vector management. Importantly, *Wolbachia*-based control strategies require controlled symbiont transfer between host species and predictable outcomes of novel *Wolbachia*-host associations. Theoretically, this artificial horizontal transfer could inflict genetic changes within transferred *Wolbachia* populations. This could be facilitated through *de novo* mutations in the novel recipient host or changes of haplotype frequencies of polymorphic *Wolbachia* populations when transferred from donor to recipient hosts. Here we show that *Wolbachia* resident in the European cherry fruit fly, *Rhagoletis cerasi*, exhibit ancestral and cryptic sequence polymorphism in three symbiont genes, which are exposed upon microinjection into the new hosts *Drosophila simulans* and *Ceratitis capitata*. Our analyses of *Wolbachia* in microinjected *D. simulans* over 150 generations after microinjection uncovered infections with multiple *Wolbachia* strains in trans-infected lines that had previously been typed as single infections. This confirms the persistence of low-titer *Wolbachia* strains in microinjection experiments that had previously escaped standard detection techniques. Our study demonstrates that infections by multiple *Wolbachia* strains can shift in prevalence after artificial host transfer driven by either stochastic or selective processes. Trans-infection of *Wolbachia* can claim fitness costs in new hosts and we speculate that these costs may have driven the shifts of *Wolbachia* strains that we saw in our model system.

## Introduction

The ubiquitous intracellular α-proteobacterium *Wolbachia pipientis* manipulates reproductive biology of many arthropod species in order to warrant its own transmission in host populations (reviewed by [1,2]). *Wolbachia* are maternally inherited and favor infected females by inducing reproductive phenotypes with cytoplasmic incompatibility (CI) as the most common ([3–5]). Besides reproductive host manipulations, *Wolbachia* can also affect nutritional and metabolic pathways of hosts ([6,7]), host development and lifespan (reviewed in [8–11]), provide protection of hosts from pathogens and parasites ([12–15]), as well as affect host mating behavior and facilitate host speciation ([16,17]).

*Wolbachia* have attracted major attention as potential novel biological control agent against the increasing threat that insect populations pose to human health and agriculture either by vectoring pathogens, or by directly causing damage to crops and life stock ([18]). *Wolbachia* could suppress insect populations through the induction of CI in a way analogous to the Sterile Insect Technique (SIT; reviewed in [19]), manipulate vectorial capacity through host lifespan-shortening effects ([11]) or vector refractoriness to pathogens in mosquitoes ([10,^15^,^20^–23]). *Wolbachia* could also be used in combination with the widely used SIT ([24]) that has encountered some problems with male fitness, mating performance, sperm size and number due to gamma irradiation of individuals ([25,26]). However, all of these *Wolbachia* applications require that (*i*) *Wolbachia* strains are transferable between diverse host systems and insect genera, (*ii*) the transferred strains are capable of inducing the expected phenotype such as CI, life shortening or resistance against pathogens, and (*iii*) transferred infection and their expected phenotypes persist stably in the novel host. So far, several authors have reported successful transfer of *Wolbachia* by microinjection from donor to recipient hosts within the same insect order, followed by confirmation of their phenotype in the novel host ([27–29,11]). The third requirement of phenotypic, and thus genomic stability has not yet been tested extensively, although artificially transferred symbiont strains can potentially experience phenotypic changes ([30]). Genotypic changes might include point mutations and genomic rearrangements triggered and facilitated by symbiont infection dynamics upon arrival and successful establishment in the novel host. In addition, pre-existing variability of *Wolbachia* in the donor host such as the presence of spurious genomic polymorphism in neotropical Drosophila species ([31]) and tsetse flies ([32]), as well as the existence of low-titer multi-infections ([33]) might affect the outcome of the artificial transfer and the stability of the expected phenotype in the new host. Recent studies have uncovered phenotypic plasticity of *Wolbachia* over evolutionary short time periods within the same host, and also upon transfer between different host backgrounds. Weeks et al. ([34]) revealed a rapid switch of *w*Ri from a parasitic ([35]) towards a more benign mutualistic state in Californian populations of *Drosophila simulans*. Adaptation of the symbiont to a novel host cell environment resulted in significant phenotypic changes of *w*MelPop when transferred between hosts ([36]). In *Drosophila melanogaster w*MelPop causes early death via over-replication in mainly nervous tissue ([37]). Trans-infection experiments caused the same lifespan reduction in *D. simulans* and *Aedes aegypti* ([38,11]). For the latter host, *w*MelPop was initially pre-adapted to *Aedes* cell lines, before its successful establishment in mosquitoes via embryonic trans-infection. Re-introduction of the *Aedes*-cell line adapted *Wolbachia* from cell lines into their native host *D. melanogaster*, however, resulted in fluctuations of maternal transmission efficiency, lower titers, and a reduced life shortening effect ([36]). These findings implicate that both host and symbiont interact dynamically and co-evolve rapidly within relatively short time periods.

It is so far unknown how genotypic integrity of *Wolbachia* is affected by recombination, genetic drift or selection after artificial host transfer. Here, we have monitored *Wolbachia* genome dynamics and population structure experimentally by utilizing two different *Wolbachia*-insect host species, *D. simulans* ([29]) and the Mediterranean fruit fly *Ceratitis capitata* ([28]). Both hosts were microinjected with *Wolbachia* of the European cherry fruit fly *Rhagoletis cerasi* ([29,28]). We tested if the transfer of *Wolbachia*, in particular of the strain *w*Cer2, *(i)* induced *de novo* structural rearrangements, and/or *(ii)* enhanced sequence polymorphism within the symbiont genome post microinjection. The potential appearance of additional *Wolbachia* subvariants, hereby designated haplotypes, that are distinctive from the reference sequence of the symbiont in its native host ([39]) can either be assigned as *de novo* mutations or ancestral haplotypes that had already persisted in the donor at low frequencies and thus had escaped earlier detection. We hypothesized that the structure of the original *Wolbachia* strain used for trans-infection plays a crucial role in the performance within the new host. It is frequently assumed that *Wolbachia* strains are monoclonal populations, and thus, only *de novo* mutations will contribute to potential diversification of the symbiont in the recipient host. In contrast, an alternative situation of *Wolbachia* strains that represent diverse populations of haplotypes such as in native tsetse flies ([32]) at varying frequencies would allow for detection of sequence variation after host transfer that was not triggered by new mutations. The trans-infection event may solely shift haplotype frequencies and thus enable detection of rare ancestral haplotypes.

To assess the potential for new structural rearrangements of *w*Cer2 in its two new host species, we took advantage of the unusual high numbers of mobile genetic elements in *Wolbachia* genomes ([40–45]) with their capacity to trigger insertions, inversions as well as ectopic recombination ([46–50]). Our analysis, however, did not reveal rearrangements. To test for sequence polymorphism, we analyzed SNP (single nucleotide polymorphism) frequency of three Multi Locus Sequence Typing (MLST) *Wolbachia* genes and traced *w*Cer2 sequence heterogeneity in original and new hosts. In the course of this in-depth analysis we found in microinjected *D. simulans* clear signs of an unexpected infection with *w*Cer1 of the original host that had remained undetected for over 150 generations. We then discuss whether heterogeneity in new hosts is caused by ancestral *Wolbachia* sequence polymorphism or arises through new mutations.

## Materials And Methods

### 2.1 Insect Lines

Fly stocks of *D. simulans* and the Mediterranean fruit fly *C. capitata* known to be infected by *w*Cer2 were used in this study. They had been microinjected with cytoplasm from the *Wolbachia*-infected cherry fruit fly *R. cerasi* thirteen ([29]) and eleven years ago ([28]). *Rhagoletis cerasi* is naturally multi-infected with up to five strains, *w*Cer1 - *w*Cer5 ([51,39]). Based on sequence analysis of MLST genes, *w*Cer1, *w*Cer2 and *w*Cer4 are A supergroup strains, *w*Cer5 a B supergroup strain, and *w*Cer3 a recombinant strain ([39,52]; http://pubmlst.org/wolbachia/). For the first host-transfer experiment, embryos of *Wolbachia*-free *D. simulans* (Nouméa TC, generated by tetracycline treatment over three generations; [53]) were injected with egg cytoplasm from Austrian *R. cerasi* donors in 200 ([29]). From this experiment, six G0 isofemales resulted in *w*Cer2 infected lines RC20, RC21, RC33, RC45, RC50 and RC78 that had to be further selected for *Wolbachia* in consecutive generations. For selection, DNA was extracted from multiple females and infection status was determined via *Wolbachia*-specific PCR. Offspring of females that tested positive for *Wolbachia* was used to proceed into the next generation via sibling mating ([29]). This selection regime was paused between G20 (2001) and G140. In 2007, selection for *Wolbachia* was continued after only five out of the six initial isofemale lines, RC20, RC21, RC33, RC45, and RC50 tested positive for *w*Cer2. The *Wolbachia* strain *w*Cer1, however, was not detected since it was considered lost between G1 and G2 ([29]). For the second host-transfer experiment, embryos of the *C. capitata* Benakeion line were injected with *Wolbachia* from *R. cerasi* from Austria and Italy (Sicily) in 2002 ([28]). This resulted in two infected *C. capitata* lines ([28]) and one of these, *Wol*Med88.6 harboring *w*Cer2, was included in our study. DNA extracts of approximately G50 post-infection were kindly provided by K. Bourtzis' laboratory (University of Ioannina, Greece). *Wolbachia*-free *D. simulans* Nouméa TC and *D. melanogaster* w^1118^ (*Wol* ^neg^) were used as negative controls, *Wolbachia*-infected DSR (*D. simulans* Riverside, California; [54]) and the *D. melanogaster* Harwich strain (*Wol* ^pos^) were used as positive control. All *Drosophila* lines were kept on standard medium at 24°C.

#### Antibiotic Treatment Of Donor And Recipient Hosts

For *Wolbachia* depletion, embryos of *R. cerasi* were transferred to larval medium containing a final concentration of 0.02, 0.025 and 0.05% (w/v) tetracycline and incubated at 24°C until reaching the third instar (L3). Antibiotic larval media and *R. cerasi* individuals were kindly provided by K. Köppler from the Center for Agricultural Technology Augustenberg (LTZ), Stuttgart, Germany. *D. simulans* RC20 and RC50 were placed on standard *Drosophila* diet containing 0.03% tetracycline for two consecutive generations before the presence of *Wolbachia* was tested.

### 2.2 Molecular Isolation And Characterization Of *Wolbachia* From *R. Cerasi* And Their Novel Hosts

#### Dna Extraction

For PCR, cloning and sequencing, high quality genomic DNA was extracted from individual pupae or adults using the Puregene DNA Purification Kit. For Southern blots, genomic DNA was extracted from individual adult flies and processed following the protocol from [55]. For *Wolbachia* depletion assays, DNA was extracted from tetracycline-treated adults of trans-infected *D. simulans* RC lines using the Puregene DNA Purification Kit (Qiagen). DNA was stored at −20°C until use.

#### Mlst-Pcr, Cloning, Sequencing And Sequence Analysis

We analyzed the frequencies of SNPs in the three *Wolbachia* MLST genes *gatB* (*WD*_0146, *w*Mel), *coxA* (*WD*_0301, *w*Mel) and *ftsZ* (*WD*_0723, *w*Mel) in donor and recipient hosts ([56],[57]). General *coxA* and *ftsZ* primer sets were used as in [56]. For *gatB*, an additional primer set amplifying a 404-bp fragment, was designed (*gatB*F 5′-gatttaaatcgtgcaggggtt-3′ and *gatB*_450R 5′-ttgaattaaatcaattttatcctgg-3′). To selectively target *w*Cer1, we used a strain-specific primer set described in [39] plus the VNTR-141 primer set from [48]. For all PCR reactions a Biometra T300 Thermocycler was used. PCR products were purified using the peqGOLD Gel Extraction Kit, inserted into the pTZ57R/T vector and then transformed into competent DH5α *Escherichia coli* cells. Clones containing the insert were Sanger sequenced at the University of Chicago Cancer Research Center (UCCRC-DSF). Sequences were analyzed using ApE plasmid editor v1.10.4 (M. Wayne Davis), CodonCode Aligner Version 2.0.3 (CodonCode Corporation) and the BLAST algorithm (www.ncbi.nlm.nih.gov).

For *D. simulans* recipients, we analyzed SNP frequencies for each line separately (RC20, RC21, RC33, RC45, and RC50) plus for the pool of all trans-infected lines (RC) in order to maximize sample size. The automated base-calling in CodonCode Aligner software detected SNPs in many clones from single individuals. In order to verify their authenticity, we visually inspected all SNPs in the corresponding chromatograms from both forward and reverse reads. All ambiguous SNPs were excluded from the final data set. Confirmed SNPs were then divided into two groups: recurrent SNPs and true singletons. Recurrent SNPs refer to nucleotide positions that were detected in independent clones in either different lines of new hosts, in *R. cerasi* clones only, or in both systems. Unique SNPs that were found in single clones only, but appeared reliable in the sequence chromatogram, were classified as singletons.

Anticipating that PCR accuracy was strongly impacted by the performance of the enzyme polymerase, we first determined the error base line of the *Taq* DNA polymerase used for all assays. We did not use a proof-reading enzyme as the Promega Go Taq® Flexi DNA Polymerase used in all experiments is one of the best non-proof-reading high quality and high performance polymerases on the market (see Promega notes available at www.promega.com). Based on re-PCR and re-sequencing of known *coxA*, *ftsZ*, and *gatB* fragments inserted into the pTZ57R/T cloning vector from independent batches of this polymerase (data not shown), we calculated the following error base lines: 0 SNPs in 4.44 kb of *coxA* (10×444 bp), 0 SNPs in 2.90 kb of *ftsZ* (10×290 bp), and 2 SNPs in 6.00 kb of *gatB* (14×429 bp). Compared to the published mean estimate for standard non-proof-reading *Taq* DNA polymerases of 0.21 SNPs/kb ([58]), our Promega Go Taq® Flexi DNA Polymerase control assay thus resulted in lower DNA polymerase error rates (0.15 SNPs/kb). We deposited 33 *coxA*, *ftsZ*, and *gatB* sequences, which represent rare allelic variants and nonsense mutations at GenBank (accession numbers KF17541-17573).

#### Restriction Fragment Length Polymorphism (rflp) Analysis Via Single Fly Southern Hybridization

We determined the structural integrity of the bacterial chromosome in the novel hosts via RFLP-analysis with highly dynamic *Wolbachia* marker sets: Insertion Sequence elements (IS) and Variable Number of Tandem Repeats (VNTRs). 0.5–1 µg genomic DNA from single flies was digested with 6U *Hin*dIII (New England Biolabs, USA) for 4 hrs at 37°C. After high-resolution vertical gel electrophoresis ([55]), gels were vacuum-blotted onto a positively charged nylon membrane (Hybond™-N+, GE Healthcare, UK). Membranes were hybridized with [α-^32^P]dCTP-labeled specific probes of IS and VNTR loci. Probes were prepared with the RediprimeTM II DNA labelling kit (GE Healthcare, UK) and exposed to high sensitivity X-ray films (Kodak, Germany). Probe primers were designed with respect to the annotated genome of *w*Mel of *D. melanogaster* (NC_002978; [40]). For transposon probing, three repeats greater than 200 bp, belonging to different IS families (*IS3*, *IS5*, and *ISNew*) in the *w*Mel genome of *D. melanogaster* were chosen ([40]). VNTR probes were *VNTR-141*, consisting of tandemly repeated 141-bp units, located at coordinates 89,003–90,332 in *w*Mel ([48]) and *VNTR-144*, consisting of 11.8 copies of a 144 bp repeat unit located at 34,727–37,210 in *w*Mel (MR, unpublished). *Hin*dIII-digested DNA of the lambda phage (New England Biolabs, USA) was utilized as size marker. Size of fragments was determined with respect to this standard, allowing the comparison of number and size of fragments between donor *R. cerasi* and new hosts *D. simulans* and *C. capitata*.

### 2.3 Ovary Screening Assay

We estimated the general fecundity cost of the artificially generated infection by comparing the ovaries of recipient and uninfected control lines of *D. simulans*. Analysis was performed between G168 and G182 post microinjection and followed the fecundity assay by [59]. Fertilized females were raised on standard food and were dissected in sterile PBS ten days after eclosion. 40 ovaries per recipient *Drosophila* line were screened. Fecundity status of ovaries was estimated according to number of mature eggs in the ovary: 0 eggs = class I; 1–2 eggs = class II; 3–9 eggs = class III; 10 and more eggs = class IV. Only eggs at stage 14 of oogenesis ([60]; indicated by dorsal appendages) were counted.

### 2.4 Statistical Analysis

For statistical analysis SPSS 16.0 and GraphPad Software (www.graphpad.com) were used. SNP frequencies were analyzed using χ^2^ with Yates Correction (2×2 contingency table); two tailed *P*-values indicated significant differences between values when <0.05. To detect potential traits of positive or negative selection, synonymous to non-synonymous substitutions per site were calculated using SNAP (Synonymous and Non-synonymous Analysis Program) provided at http://www.hiv.lanl.gov. This program uses the Nei-Gojobori corrected path counting method that adjusts for counts via Jukes-Cantor plus the weighting of pathways from one codon to another according to an equi-probable model for each possible codon-to-codon path ([61]).

## Results

### 3.1 Conserved *Wolbachia* Genome Synteny After Artificial Transfer Into Novel Recipient Hosts

We analyzed the genome synteny of artificially transferred *w*Cer2 infection from *R. cerasi* into *D. simulans* and *C. capitata* with five marker probes for RFLP mapping (**Figure S2**). Although IS and VNTRs had earlier been reported as hypervariable entities of *Wolbachia* genomes, we did not detect any structural re-arrangements for the five tested loci in *w*Cer2 of the novel hosts (**Figure S2** and **Data S1**).

### 3.2 Snp Frequency In *Gatb*, *Coxa*, And *Ftsz* Of Recipient Host Populations

Prior to all sequencing experiments, we determined the base line error rate during polymerase chain reaction of the *Taq* polymerase used in our lab. Consequently, we analyzed SNP frequencies in three *Wolbachia* loci (*gatB*, *coxA*, and *ftsZ*) in trans-infected *D. simulans* and *C. capitata*. For *gatB*, we sequenced and analyzed 29.1 kb: 12×404 bp from each of RC20, RC21, RC33, RC45, RC50 of *D. simulans*, plus 12×404 bp from *Wol*Med88.6 of *C. capitata* (**Table S1**). In total, 38 SNPs randomly dispersed along the 404 bp *gatB* gene fragment (**Table 1**) were detected. Out of these 38 SNPs, 6 (16%) were found recurrently, and 32 (84%) were singletons. Recurrent SNPs were SNP-11 (2×); SNP-42 (4×); SNP-93 (2×); SNP-186 (2×); SNP-250 (2×), and SNP 253 (2× ochre mutation, *i.e.*, CAA to TAA, occurring in RC of *D. simulans* and in *Wol*Med88.6; **Table 1**). Overall SNP frequency for *gatB* of *w*Cer2 was 1.01 SNPs/kb (**Table 2A**). For *coxA* of *w*Cer2 we analyzed 16.4 kb (37×444 bp amplicon) with an average SNP-frequency of 1.64 SNPs/kb (**Table 2B**). For *ftsZ*, we analyzed 7.6 kb of *w*Cer2 (16×478 bp amplicon) with an average SNP-frequency of 0.78 SNPs/kb (**Table 2C**). Overall SNP-frequencies for all three genes were significantly higher, *i.e.*, 5–11fold, than the error base line we had determined before for the *Taq* polymerase (compare frequencies of 0.78, 1.01, and 1.64 SNPs/kb to 0.15 SNPs/kb from Taq polymerase).

**Table 1:** Variable nucleotide positions in *gatB* (A) and amino acid positions in GATB (B) of *w*Cer2.

**variable nucleotide positions in ** ***gatB*** ** of ** ***w*** **Cer2**	3	11	16	31	32	36	42	61	72	88	89	93	102	112	121	149	186	195	206
**consensus**	**T**	**A**	**G**	**C**	**T**	**C**	**T**	**A**	**A**	**T**	**A**	**T**	**T**	**A**	**A**	**C**	**T**	**A**	**A**
***w*** **Cer2 ** ***R. cerasi***	**.**	**.**	**.**	**.**	**.**	**.**	C	G	G	**.**	**.**	**.**	**.**	**.**	**.**	T	**.**	G	**.**
***w*** **Cer2 RC21**	**.**	**.**	**.**	**.**	**.**	**.**	**.**	**.**	**.**	**.**	**.**	**.**	**.**	G	**.**	**.**	**.**	**.**	**.**
***w*** **Cer2 RC20**	**.**	**.**	**.**	**.**	**.**	**.**	C	**.**	**.**	**.**	**.**	C	C	**.**	T	**.**	C	**.**	**.**
***w*** **Cer2 RC33**	**.**	**.**	**.**	**.**	A	**.**	**.**	**.**	**.**	**.**	G	**.**	**.**	**.**	**.**	**.**	**.**	**.**	**.**
***w*** **Cer2 RC45**	C	**.**	**.**	**.**	**.**	**.**	**.**	**.**	**.**	**.**	**.**	**.**	**.**	**.**	**.**	**.**	**.**	**.**	**.**
***w*** **Cer2 RC50**	**.**	G	**.**	**.**	**.**	**.**	**.**	**.**	**.**	C	**.**	**.**	**.**	**.**	**.**	**.**	**.**	**.**	**.**
***w*** **Cer2 ** ***Wol*** **Med88.6**	**.**	**.**	A	T	**.**	A	**.**	**.**	**.**	**.**	**.**	**.**	**.**	**.**	**.**	**.**	**.**	**.**	G
**SNP-Frequency**	singleton	2× in RC	singleton	singleton	singleton	singleton	3× in RC, 1× in *R. cerasi*	singleton	singleton	singleton	singleton	2× in RC	singleton	singleton	singleton	singleton	2× in RC	singleton	singleton
**variable nucleotide positions in ** ***gatB*** ** of ** ***w*** **Cer2**	221	226	238	250	253	257	288	305	321	323	324	343	350	354	355	367	371	390	398
**consensus**	**T**	**C**	**T**	**A**	**C**	**T**	**T**	**T**	**A**	**A**	**A**	**A**	**A**	**A**	**A**	**T**	**T**	**A**	**A**
***w*** **Cer2 ** ***R. cerasi***	C	**.**	**.**	**.**	**.**	**.**	**.**	**.**	G	**.**	G	**.**	**.**	**.**	**.**	**.**	**.**	**.**	**.**
***w*** **Cer2 RC21**	**.**	**.**	**.**	**.**	**.**	**.**	**.**	**.**	**.**	**.**	**.**	**.**	**.**	**.**	**.**	**.**	**.**	**.**	**.**
***w*** **Cer2 RC20**	**.**	T	**.**	**.**	**.**	**.**	**.**	C	**.**	G	**.**	G	**.**	**.**	**.**	**.**	**.**	**.**	**.**
***w*** **Cer2 RC33**	**.**	**.**	**.**	G	**.**	**.**	**.**	**.**	**.**	**.**	**.**	**.**	**.**	**.**	**.**	C	**.**	**.**	**.**
***w*** **Cer2 RC45**	**.**	**.**	C	**.**	**.**	C	C	**.**	**.**	**.**	**.**	**.**	**.**	G	**.**	**.**	**.**	C	**.**
***w*** **Cer2 RC50**	**.**	**.**	**.**	**.**	T	**.**	**.**	**.**	**.**	**.**	**.**	**.**	**.**	**.**	G	**.**	C	**.**	**.**
***w*** **Cer2 ** ***Wol*** **Med88.6**	**.**	**.**	**.**	G	T	**.**	**.**	**.**	**.**	**.**	**.**	**.**	G	**.**	**.**	**.**	**.**	**.**	G
**SNP-Frequency**	singleton	singleton	singleton	1× RC, 1× in *Wol*Med88.6	1× RC, 1× in *Wol*Med88.6	singleton	singleton	singleton	singleton	singleton	singleton	singleton	singleton	singleton	singleton	singleton	singleton	singleton	singleton
**variable amino acid positions in GATB of ** ***w*** **Cer2**	1	4	6	11	11	12	14	21	24	30	30	31	34	38	41	50	62	65	69
**consensus**	**A**	**E**	**V**	**L**	**L**	**L**	**S**	**M**	**R**	**Y**	**Y**	**I**	**C**	**M**	**G**	**F**	**R**	**I**	**N**
**aa changes in all RC lines and ** ***Wol*** **Med88.6**	**.**	**G**	**I**	**F**	**R**	**.**	**.**	**V**	**G**	**H**	**C**	**.**	**.**	**V**	**.**	**S**	**.**	**M**	**S**
**variable amino acid positions in GATB of ** ***w*** **Cer2**	74	76	80	84	85	86	96	102	107	108	108	115	117	118	119	121	124	130	133
**consensus**	**I**	**Q**	**D**	**R**	**E**	**I**	**S**	**F**	**G**	**K**	**K**	**L**	**D**	**A**	**S**	**Y**	**F**	**L**	**E**
**aa changes in all RC lines and ** ***Wol*** **Med88.6**	**T**	*	**.**	**G**	*	**T**	**.**	**S**	**.**	**R**	**.**	**E**	**E**	**.**	**G**	**H**	**S**	**F**	**G**

**A**) Position 1 in the presented 404 bp fragment corresponds to position 981 of the full *gatB* locus of *w*Ri infecting *Drosophila simulans* Riverside (GenBank accession number CP001391). Aa position 1 in (**B**) corresponds to aa position 148 of the full GATB protein of *w*Ri (protein ID:ACN94961.1). Frequency of SNP indicates which SNPs are singletons or occur recurrently in what host system. Nonsense mutations leading to a stop codon are indicated by asterisks. Abbreviations: aa amino acid.(

**Table 2:** SNP frequencies in *w*Cer of *R. cerasi* and *de novo* hosts.

A							
no	*gatB* of wCer	bases	SNP-	SNP-	assay	*P* value^c^	*P* value^d^
			frequency^a^	frequency^b^			
1	*w*Cer1 of *R. cerasi*	9696	0.52	*nd*			*nd*
2	*w*Cer2 of *R. cerasi*	8888	1.01	0.73	1 *vs.* 2	0.3346	0.8854
3	*w*Cer1 of RC	1212	2.48	*nd*	1 *vs.* 3	0.0703*	*nd*
4	*w*Cer2 of RC	29088	1.01	0.73	2 *vs.* 4	0.6057	0.6152
5	RC20	4848	2.68	2.40	4 *vs.* 5	0.0188*	0.0109*
6	RC33	4848	0.83	0.55	4 *vs.* 6	0.2103	0.6386
7	RC45	4848	1.24	0.96	4 *vs.* 7	0.9097	0.8714
8	RC50	4848	1.03	0.75	4 *vs.* 8	0.9718	0.9391
9	RC21	4848	0.00	−0.28	4 *vs.* 9	0.0622*	*nd*
10	*Wol*Med88.6	4848	1.65	1.37	4 *vs.* 10	0.4453	0.3824

B							
no	*coxA* of *w*Cer	bases	SNP-	SNP-	assay	*P* value^c^	*P* value^d^
			frequency^a^	frequency^b^			
1	*w*Cer1 of *R. cerasi*	5772	0.69	*nd*			*nd*
2	*w*Cer2 of *R. cerasi*	888	0.00	*nd*	1 *vs.* 2		*nd*
3	*w*Cer1 of RC plus	4911	1.63	*nd*	1 *vs.* 3	0.2510	*nd*
	*Wol*Med88.6						
4	*w*Cer2 of RC	16428	1.64	*nd*	2 *vs.* 4	0.4406	*nd*

C							
no	*ftsZ* of *w*Cer	bases	SNP-	SNP-	assay	*P* value^c^	*P* value^d^
			frequency^a^	frequency^b^			
1	*w*Cer1 of *R. cerasi*	10516	1.14	*nd*			*nd*
2	*w*Cer2 of *R. cerasi*	10038	1.10	*nd*	1 *vs.* 2	0.9288	*nd*
3	*w*Cer1 of RC	*nd*	*nd*	*nd*	*nd*	*nd*	*nd*
4	*w*Cer2 of RC	7648	0.78	*nd*	2 *vs.* 4	0.6771	*nd*

Frequencies were calculated for (**A**) *coxA*, (**B**) *ftsZ*, and (**C**) *gatB*. Column ‘bases’ gives the total number of sequenced bases.

^a^ SNP-frequency per kilobase;

^b^ SNP-frequency per kilobase minus calculated error base line (0.28/kb) of *Taq* polymerase;

^c^ two-tailed *P* values from χ^2^ calculations with Yates Correction (2×2 contingency table) for ^a^;

^d^ two-tailed *P* values from χ^2^ calculations with Yates Correction (2×2 contingency table) for ^b^.

Abbreviations: *nd* not determined.

### 3.3 Uncovering Cryptic Co-Infection With The *W*cer1 Strain

**Figure 1:**
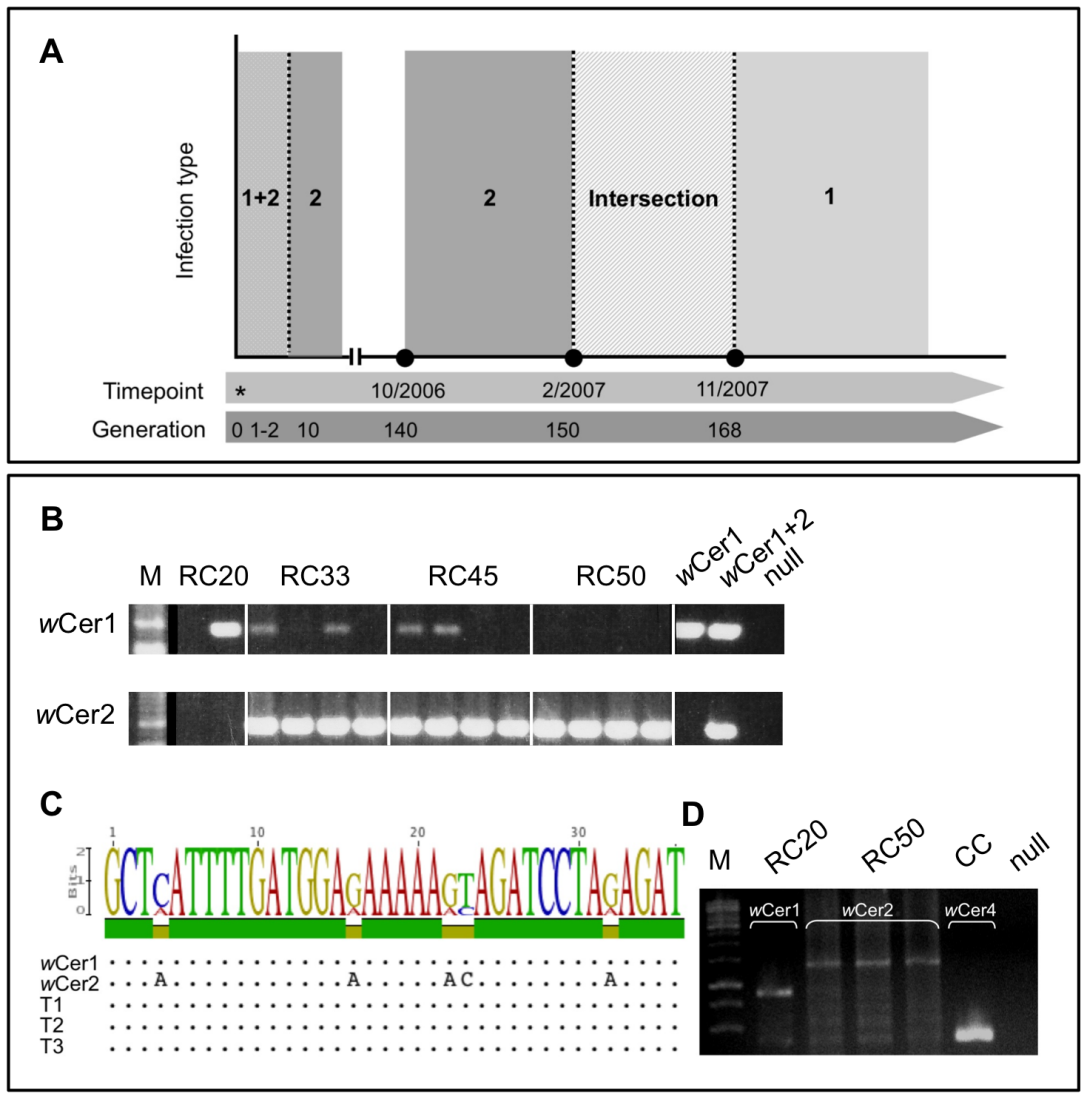
Cryptic co-infection with *w*Cer1 in *w*Cer2 carrying trans-infected lines. (**A**) Switch of strain prevalence from *w*Cer2 to *w*Cer1 in RC20. Asterisk represents time point of line establishment via microinjection in 200. Generations are indicated on x-axis. (**B**) Presence of *w*Cer1 in random samples of RC lines determined via strain-specific *wsp* PCR. First RC20 sample seems to carry *Wolbachia* at extremely low density below detection limit of *wsp* PCR whereas the other one gives a bright band with *w*Cer1-specific *wsp* primer set. DNA extracted from adult *Rhagoletis cerasi* served as positive controls (*w*Cer1 and *w*Cer1+2). (**C**) Random 36-bp fragment of the general *wsp* amplicon showing diagnostic *w*Cer1/*w*Cer2 sites. (**D**) Differentiation between *w*Cer1, *w*Cer2 and *w*Cer4 *Wolbachia* using VNTR-141 PCR. Abbreviations: CC *C. capitata*, M DNA size marker, T1-3 trans-infected RC line sample.

Unexpectedly, we detected sequence traces of *w*Cer1 in three lines of microinjected *D. simulans*, although this strain had previously been considered as lost from the microinjected lines between G1 and G2 ([29]; and **Figure 1A**). To confirm the presence of *w*Cer1 independently, we performed PCR analyses utilizing primer sets specifically targeting *wsp* of either *w*Cer1 or *w*Cer2, respectively ([39]; **Figure 1B**). According to the signal intensity of the *wsp* amplicon during electrophoresis, RC20 harbored *w*Cer1 at high densities whereas *w*Cer2 could no longer be tracked (**Figure 1B**). *Wsp* sequence reads from RC20 revealed exclusively the *w*Cer1 haplotype (**Figure 1C**). In RC33 and RC45, we traced *w*Cer1 co-infecting *w*Cer2 (**Figure 1B**). RC50 showed no signals of *w*Cer1 but of a single infection with *w*Cer2 (**Figure 1B**). In addition to *wsp*, the presence of *w*Cer1 in RC20 and its absence in RC50 was confirmed by the diagnostic VNTR-141 locus via PCR (**Figure 1D**).

After verification of the *w*Cer1 sequences we analyzed the SNP frequency of this strain using *gatB* and *coxA* in a small sample of new hosts. We tested 1,212 bp for *gatB* with an average SNP-frequency of 2.48 SNPs/kb (**Table 2A**). For *coxA* we sequenced 4.9 kb (3×305 bp amplicons from RC20 and 9×444 bp from *Wol*Med88.6 and RC20 and RC33; **Table 2B**) with an average SNP frequency of 1.63 SNPs/kb. SNP frequencies for *ftsZ* of *w*Cer1 in novel hosts were not determined due to the low coverage (**Table 2C**).

Regarding the heterogeneity detected in *w*Cer, we hypothesized that the SNPs can (*i*) either represent ancestral, hidden sequence polymorphism, *i.e.*, multiple *Wolbachia* haplotypes already present in the donor host, or (*ii*) have arisen *de novo* following microinjection. To test this hypothesis, we compared SNP frequencies in the novel hosts with the frequencies in the donor of the *Wolbachia* strains. Since original donor specimens for the microinjection experiments into *D. simulans* and *C. capitata* were not kept as voucher material, we sequenced *gatB*, *coxA*, and *ftsZ* from a broader representative range of other *R. cerasi* populations across Europe instead.

### 3.4 Snp Frequency Baseline In *Gatb*, *Coxa*, And *Ftsz* Of Donor And Comparison With Recipient Host Populations

We analyzed *Wolbachia* SNP frequencies from *R. cerasi* samples collected all over Europe ([39]). In total, we sequenced 8.9 kb (22×404 bp amplicons) of *w*Cer2 *gatB* from *R. cerasi* derived from individuals from more than ten collection sites across Europe (**Table S2 and Table S4**). As shown in **Table 2A** the average SNP frequency for *gatB* of *w*Cer2 from the donor *R. cerasi* was 1.01/kb (9 variable positions in 8.9 kb). Comparing these data with those of the novel hosts, we did not observe an increase in SNP-frequencies per kb of *w*Cer2 *gatB* in the recipients (**Table 2**). Only one (SNP-42) out of the 38 SNPs, however, determined in *w*Cer2 *gatB* of the novel hosts, occurred also in the original donor *R. cerasi*.

Since we unexpectedly detected *w*Cer1 in three trans-infected host lines we included this *Wolbachia* strain in our SNP analyses (however at a lower coverage). For *gatB* of *w*Cer1, we sequenced 9.7 kb consisting of a 24×404 bp amplicons data set and calculated an average SNP-frequency for *gatB* of *w*Cer1 in *R. cerasi* that was not statistically significant from *w*Cer2 in *R. cerasi* (0.52 *vs.* 1.01; *P* = 0.3346; **Table 2A**). The average SNP-frequency in *gatB* of *w*Cer1 in novel hosts lines was also not higher than in the native host *R. cerasi* (2.48 *vs.* 0.52; *P* = 0.0703), but the small sample set (1,212 bp) impeded statistical testing and thus did not allow a reliable comparison between both *w*Cer 1 and *w*Cer2.

For the *coxA* locus, we sequenced 5.7 kb of *w*Cer1 (13×444 bp amplicons), and 888 bp of *w*Cer2. Average SNP-frequencies for *w*Cer1 and *w*Cer2 infections were rather low (0.69 SNPs/kb and 0 SNPs/kb, respectively; **Table 2B**). It must be taken into account that the data set for *w*Cer2 consisted only of two clone reads and can thus not be regarded as highly representative.

For the *ftsZ* locus, we sequenced 10.5 kb of *w*Cer1 (23×478 bp amplicons) and 10 kb of *w*Cer2 (21×478 bp amplicons). At this locus SNP frequencies of the two *Wolbachia* strains were quite similar with 1.14 SNPs/kb and 1.10 SNPs/kb for *w*Cer1 and *w*Cer2, respectively (**Table 2C**). One SNP in *ftsZ* of *w*Cer1 resulted in a transversion from guanine to thymine in the first position of the consensus triplet GGA (Gly), thus introducing the stop codon TGA to the sequence. We detected this stop codon twice and independently in *ftsZ* of *w*Cer1.

### 3.5 Presence Of Stop Codons In *W*cer1 And *W*cer2 Of Essential *Wolbachia* Genes In Both Donor And Recipient Host Populations

The recurrent finding of SNPs causing nonsense mutations in essential *Wolbachia* housekeeping genes was highly unexpected and hence considered with extreme caution. However, similar to the ochre mutation in *gatB* of *w*Cer2 in *D. simulans* and *C. capitata* (see 3.2), we found additional nonsense mutations in *coxA* and *ftsZ* of *w*Cer1 *Wolbachia* (**Table 3**). A recurrent SNP (4×) in *fts*Z of *w*Cer1 of its native host *R. cerasi* caused a transition of guanine to thymine in the first position of a GGA triplet in *w*Cer1 (opal mutation). Finally we also uncovered an ochre mutation in *coxA* of *w*Cer1 in recipient line RC20 but as a singleton only (**Table 3**). In order to test whether such *Wolbachia* pseudogenes might stem from translocations onto the host chromosome, tetracycline-treated individuals of RC20, RC50, and *R. cerasi*, plus their corresponding untreated controls were tested via *gatB* PCR for the presence of potential nuclear *Wolbachia* copies. As shown in **Figure S1**, both recipient lines lost the *gatB* PCR signal after two generations of antibiotic treatment, which makes a lateral gene transfer event unlikely. Hence alternative scenarios will be necessary for explaining these counter intuitive findings, *i.e.*, the persistence of nonsense mutations in essential *Wolbachia* genes (see discussion).

**Table 3:** Stop codons in *gatB*, *coxA*, and *ftsZ* of *w*Cer1 and *w*Cer2 *Wolbachia*.

Line	Gene	Position	Mutation	*Wol*-infection
			**C**AA = consensus	*w*Cer2
RC20	*gatB*	226/404	**T**AA (ochre)	*w*Cer2
RC50	*gatB*	253/404	**T**AA (ochre)	*w*Cer2
*Wol*Med88.6	*gatB*	253/404	**T**AA (ochre)	*w*Cer2
			**A**AA = consensus	*w*Cer1
RC20	*coxA*	22/444	**T**AA (ochre)	*w*Cer1
			**G**GA = consensus	*w*Cer1
F37 eastern Sicily	*ftsZ*	25/478	**T**GA (opal)	*w*Cer1
F38 eastern Sicily	*ftsZ*	25/478	**T**GA (opal)	*w*Cer1
F40 western Sicily	*ftsZ*	25/478	**T**GA (opal)	*w*Cer1
F42 western Sicily	*ftsZ*	25/478	**T**GA (opal)	*w*Cer1

Lane three lists the position of the mutation corresponding to the size of the amplified MLST-gene fragment. Lines F37 to F42 represent *R. cerasi* individuals from different populations sampled in Sicily, Italy.

### 3.6 Trans-Infection With *W*cer Is Costly For The Recipient

**Figure 2:**
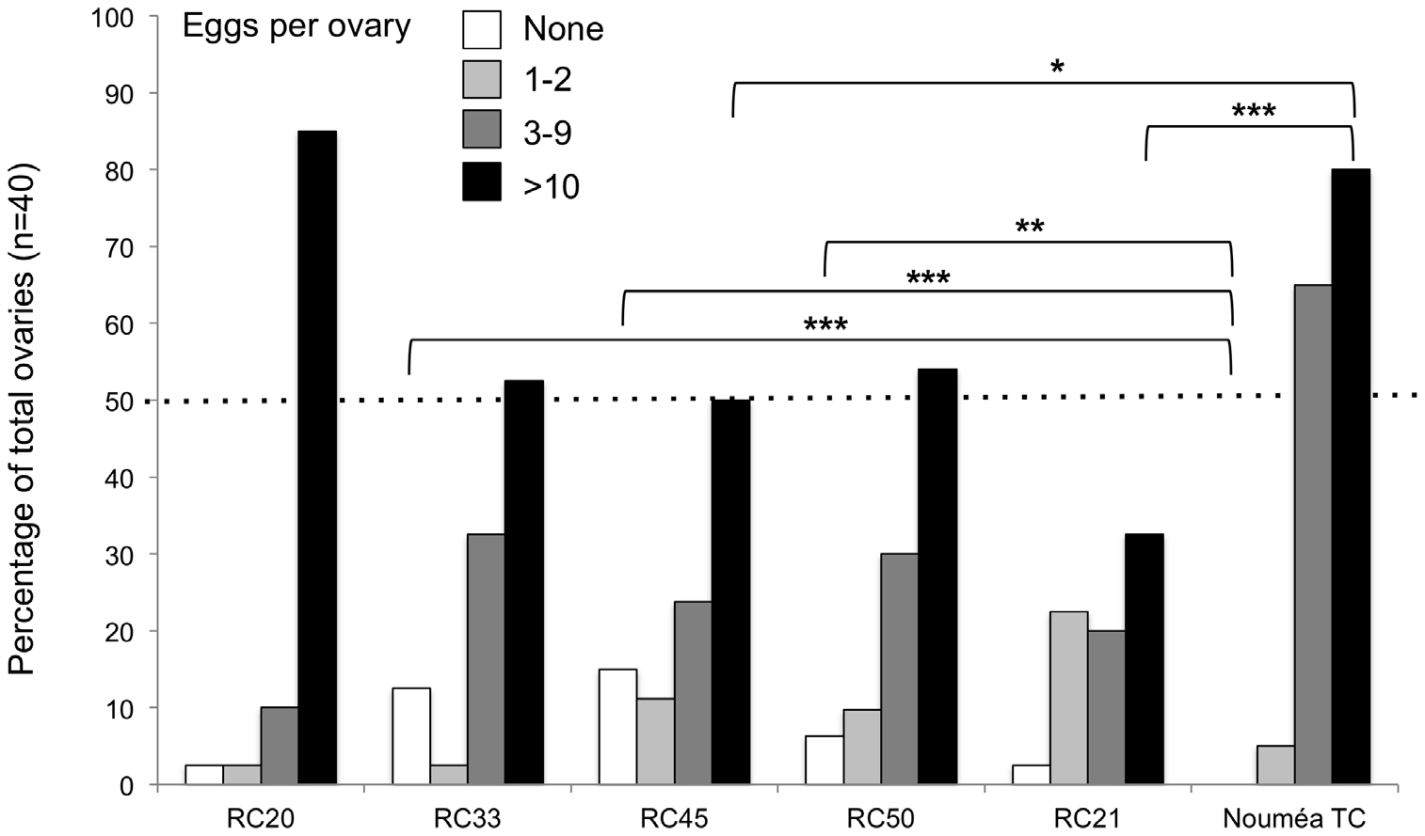
Ovary screen in trans-infected RC lines. Size of ovaries was compared on basis of number of mature eggs in one ovary. Bars represent ovary size per line determined for each ovary class: white is class I with no mature eggs; light grey class II (1–2 eggs); dark grey class III (3–9 eggs); and black is class IV with 10 or more eggs. Y-axis shows percent of ovaries per class; x-axis shows RC lines plus the *Wolbachia*-unifected *D. simulans* Nouméa TC control. Significances based on two-tailed *P* values from Fisher's exact test are indicated by asterisks.

Between G168 and G182 post-microinjection we performed an ovary screening assay ([59]) to estimate the costs of establishing an artificial infection with *Wolbachia*. Based on the number of mature eggs per ovary, individuals were sorted into the four fecundity classes. In total, we screened 40 individuals per *D. simulans* line including 40 individuals from *Wolbachia*-uninfected *D. simulans* Nouméa TC, which had been used as recipient for the microinjection. As shown in **Figure 2**, percentage of individuals in both extreme classes, I and IV, varies highly among new hosts. Compared to 80% in the control (Nouméa TC), RC21 shows only 32.5% of individuals in class IV, suggesting poor fecundity (*P* = 0.004***). Except for RC20, also the other new hosts exhibited reduced number of mature eggs (ranging from 52.5 to 60% in class IV) pointing towards a fitness cost effect related to the new symbiont infection. Interestingly, in RC20 percentage of class IV ovaries is higher than in all other new hosts and even slightly higher then in the control (85% vs. 80%). This might represent a positive correlation of fecundity with *Wolbachia* infection in this special case, but would require further testing for verification (see discussion).

## Discussion

### 4.1 No Traces Of Structural Re-Arrangements Detectable By Means Of *Wolbachia* Mobile Genetic Elements Upon Artificial Transfer Into Novel Recipient Hosts

We looked at two highly informative VNTR markers and three *Wolbachia* transposons (*IS3*, *IS5*, and *ISNew*) via Southern hybridization, and thus covered at least 30 loci dispersed in the *w*Cer2 genome. We did, however, not detect any transpositions in these *loci*. Recent studies demonstrated that *Wolbachia* carry a high percentage of functional transposable elements that can display transpositional activity during short term *Wolbachia* evolution ([47–49]). A study of polymorphism in IS element insertions sites and VNTRs of *w*Mel revealed that the previously assumed homogeneous *Wolbachi*a infection of *D. melanogaster* is a set of different variants, such as *w*Mel and *w*MelCS ([48,62]). However, given that so far 20 major families of insertion sequences have been classified from 171 bacterial and archeal species ([63,64]) we cannot rule out that any transposition effect occurred in our study system with any other mobile elements. Thus, as a next step, whole genome sequencing of *w*Cer will be the most adequate strategy to obtain sufficient information on potential structural rearrangements in the symbiont genome upon transfer into recipients.

### 4.2 *Wolbachia* Strains Are Most Likely Bacterial Populations Of Diverse Haplotypes With Shifting Frequencies

We determined SNP frequencies for *gatB*, *ftsZ*, and *coxA*, between native and recipient hosts and did not detect any SNP differences that could have arisen after microinjection. The SNP-frequencies in *ftsZ*, and *gatB* of *w*Cer2 in the native host *R. cerasi* were with approximately only 1 SNP/kb low. For *coxA*, the frequency was even lower (0 SNPs/kb) but this might not be highly representative since we tested a very small sample size. In the recipient hosts *D. simulans* and *C. capitata*, we determined SNP-frequencies in *w*Cer2 that were not accelerated compared to *R. cerasi* (see **Table 2**). Although not statistically significant, we observed a trend towards an increase in SNP-frequency, at least in *gatB* of one of the recipients (RC20), at the time when *w*Cer2 was unambiguously present). We calculated the ratio of the number of non-synonymous substitutions per non-synonymous site (d_N_) to the number of synonymous substitutions per synonymous site (d_S_) for *gatB* of *w*Cer2, a general indicator for selective pressure acting on protein-coding genes. This ratio for *gatB*, in *R. cerasi* and RC lines (**Table S3**) corroborated our finding that SNP-frequencies did not change upon arrival of *Wolbachia* in the new host systems.

Similar to *w*Cer2, we found overall low SNP-frequencies in *gatB*, *coxA*, and *ftsZ* of *w*Cer1 in the native host *R. cerasi*, ranging from 0.5 to 1.1 SNP/kb. For the trans-infected hosts, we only analyzed the frequency of *gatB* and *coxA*, not for *ftsZ*. Both of these data sets are rather small and thus still inconclusive. Unexpectedly, we revealed cryptic double infections with *w*Cer1, but this *Wolbachia* strain persisted in lower densities than the predominant *w*Cer2. Since the focus of this study was from its onset primarily on wCer2 and not wCer1, a more extensive analysis of *w*Cer1 sequences deriving from the trans-infected host systems was not carried out. Our *coxA* sequence data, however, did not indicate an increased *w*Cer1 SNP-frequency in the *de novo* hosts. In particular, in *w*Cer1-*gatB*, we did not find a statistically significant increase in the SNP frequency within RC compared to *R. cerasi*.

As shown in **Table 1**, the variable sites we detected in *gatB* of *w*Cer2 were either present in the native and/or in the trans-infected hosts. This finding raises the question whether the observed polymorphism represented *de novo* mutation events or ancestral cryptic infection polymorphism. [65] recently showed that new *Wolbachia* haplotypes might be generated by point mutations in outer membrane proteins. In our study 68% of the SNPs revealed in *w*Cer2 of *gat*B (26/38) were not detected in the donor of the infection but exclusively in the trans-infected hosts, suggesting *de novo* mutation events. Our PCR, cloning and Sanger sequencing based approach resulted in relatively low sample size, so that this study is not sensitive enough to rule out the existence of rare haplotypes in the donor host. Deep-sequencing strategies of donor and recipient with much higher coverage will be of pivotal interest to finally uncover potential *de novo* mutation rates of the endosymbiont upon artificial host switch. An alternative reason for our failure of detecting shared SNPs in both donor and recipients is genetic drift. Through drift effects, allele frequencies change and this may result in the loss of certain haplotypes followed by consequent reduction of genetic variation within a population. As a third explanation, selection for certain, beneficial haplotypes can be envisaged. This would lead to subsequent fixation of these haplotypes and loss of others.

We found, however, that SNP-42 in *gatB* of *w*Cer2 (**Table 1**) occurred recurrently in both donor and recipient systems (RC20), clearly suggesting an ancestral origin. It is possible that the mutation in position 42 in the 404-bp *gat*B fragment represents a rare haplotype of *w*Cer2 that coexists with the canonical haplotype in the *R. cerasi* donor ([39]; this study). Moreover, SNP-250 of *gatB* of *w*Cer2 that results in the replacement of Arg with Gly (**Table 1**) was found in both heterologous hosts *D. simulans* and *C. capitata*, but not in the donor. This situation is similar to SNP-253 of *gatB* of wCer2 that results in a nonsense mutation (see below). SNP-11, SNP-93 and SNP-186 provide three additional cases for the existence of distinctive *gatB* haplotypes of the *w*Cer2 infection since all three were repeatedly isolated from hosts that were independently microinjected (**Table 1**). Hence the *w*Cer2 infection cannot be considered monoclonal but a bacterial population of diverse haplotypes at varying frequencies. This idea is supported by the recent finding from Symula and colleagues who proposed the existence of high *Wolbachia* sequence variation between and within individuals of the tsetse fly *Glossina fuscipes fuscipes* ([32]).

If *w*Cer2 infection is a population of haplotypes, rare haplotypes within this population might be difficult to detect. Any change in the structure of this bacterial population, however, massively impacts the frequency of haplotypes. Events that impact the population structure as well as population size in such a crucial way are referred to as bottleneck events. The artificially transfer of *w*Cer2 from its native host *R. cerasi* into two new hosts, was such a bottleneck event and thus manipulated the structure of the original *w*Cer2 population. We argue that this resulted in the shift of haplotype frequencies in the trans-infected lines. It is likely that the polymorphism that we observed in *gatB* upon arrival in *D. simulans* and *C. capitata* represented rare haplotypes that already persisted in the ancestral and native *w*Cer2 population of *R. cerasi* and are only detectable after the artificially induced bottleneck scenarios.

### 4.3 *Wolbachia* Strains Accumulate Nonsense Mutations Upon Arrival In New Hosts

We tested if SNP frequency in *Wolbachia* genes is increased after microinjection, thus suggesting relaxation of purifying constraints on these genes. Our results did not explicitly support such an effect although a slight trend towards diversifying selection was still observed (see 4.2). We revealed, however, that three out of 38 SNPs (8%) detected in *gat*B of *w*Cer2 introduced novel pre-mature stop codons caused by in-frame ochre mutations, *i.e.* a transition of cytosine to thymine in the first position of a CAA triplet. SNP-226 was found uniquely in transinfected RC20, whereas SNP-253 was found in both recipient hosts independently, *i.e. D. simulans* (line RC50) and *C. capitata* (line *Wol*Med88.6). Novel stop codons were not restricted to *w*Cer2 since we also traced them in *coxA* and *ftsZ* of *w*Cer1. SNP-22 in *coxA* of *w*Cer1 was found uniquely in line RC20. In contrast, SNP-45 in *ftsZ* of *w*Cer1 seemed to be of ancestral origin, occurring in Sicilian *R. cerasi* populations only.

A growing body of empirical evidence has demonstrated that *Wolbachia* genes and even complete genomes are being transferred onto insect host chromosomes ([66–69]). Such lateral gene transfer events can explain the accumulation of nonsense mutations when fully functional copies of these genes are still present in the symbiont genome. In order to test for lateral gene transfer, we cleared the recipient lines with antibiotics. Symbiont genes transferred into the host genome would be not be affected and thus still detectable. Our screen of treated RC lines did not indicate any gene transfer event, suggesting that the detected nonsense mutations are present in cytoplasmic *Wolbachia*.

In total, we found two types of nonsense mutations in recipient and donor hosts, ochre and opal. In the recipient systems, we revealed two in-frame ochre mutations in *w*Cer2 of *gat*B, and two in-frame opal mutations in *w*Cer1 of *coxA*. In the original donor system we determined one in-frame mutation in *fts*Z of *w*Cer1. Mutant tRNA is able of suppressing some stop codons in *E. coli* ([70,71]), and allele-specific super-suppressor mutants have been reported for the yeast *Saccharomyces cerevisiae* ([72]). We performed a PCR-based pilot screen for *Wolbachia* candidate tRNA suppressor mutants that would be able to rescue both ochre mutations in w*C*er1 but did not find any (data not shown). It can hence not yet be explained why unexpectedly high frequencies of nonsense mutations occurred in two *Wolbachia* housekeeping genes of both *Wolbachia* strains. We propose, however, several ideas that might explain the compensation of these mutations. First, the ‘codon capture model’ allows a bacterial codon that has fallen to low frequencies to be reassigned without triggering fitness implications ([73,74]). In the case of the ochre mutation in *w*Cer2, TGA would be re-coded into a synonym and hence not affect protein length. Alternatively, in concert with our theory of co-existing *w*Cer2 haplotypes in the population of one single host, it is possible that one haplotype carries the nonsense mutation whereas another one does not. A fully functional *w*Cer2 haplotype could then potentially compensate the mutation in the non-functional haplotype. Generally, bacteria are assumed to be monoploid *i.e.*, they carry only one copy of a circular chromosome. Recent publications have demonstrated that this is not necessarily case. [75] have shown that Neisseira gonorrhoeae are polyploidy and carry three genome copies in average. [76] have added striking new findings by stating that monoploidy is not typical for bacteria. In contrast, polyploidy is very common in proteobacteria with up to even 20 genome copies. Following these interesting findings, it might be possible that *Wolbachia* also contain more than one genome copy per cell. If those copies are different, *i.e.*, one carries the mutation and the other does not, the *Wolbachia* sequence polymorphism we detected in this study can be explained. Alternatively, but highly unlikely, the formation of paralogues via intrachromosomal duplications of the three *Wolbachia* genes *coxA*, *ftsZ* and *gatB* can be employed. Finally, it could also be speculated that alternative genetic codes might support compensating the nonsense mutations. Contrasting to the bacterial code, the TAA triplet does for example not lead to a termination signal in the ciliate, dasycladacean and Hexamita nuclear genomes as well as in the alternative flatworm mitochondrial code. The TGA codon can be compensated by even nine alternative codes (source: www.ncbi.nlm.nih.go). So far, we do not know how stably these mutant haplotypes are maintained within the *w*Cer population but an ongoing deep sequencing project of the *w*Cer2 genome will allow us to screen, in detail, for the presence and maintenance of these mutations.

### 4.4 Maintenance And Frequency Shifts Of Diversity After Trans-Infection

Multiple *Wolbachia* strains can coexist within single host individuals *e.g.* ants ([77]) and tephritid fruitflies ([78,39]). However, simultaneous persistence of more than one *Wolbachia* strain within a single host raises the question as to whether inter-strain competition for survival and stable persistence does occur. In the case of the observed strain replacement in RC20, inter-strain competition between *w*Cer1 and *w*Cer2 is expected. We collected evidence that the initially common *w*Cer2 was no longer traceable in G168 after the trans-infection event, supporting the idea of strain replacement following inter-strain competition. Recent studies demonstrated that *Wolbachia* infections can occur at extreme low titer levels. The persistence of such natural low titer *Wolbachia* infections have been reported in bark beetles ([33]), neotropical Drosophila species ([17]), aphids ([79]), *D. simulans* ([80]) and tsetse flies ([81]). We hence assume that *w*Cer2 density is extremely variable, making detection of the symbiont impossible even by using highly sensitive techniques. However, the artificial double infection in the new host background might have triggered ongoing inter-strain competition. An explanation for *w*Cer1 displacing prevalent *w*Cer2 in RC20 might be a negative symbiont-host productivity correlation (see 4.5). Beaumont and colleagues recently reported on the experimental evolution of bet hedging strategies in bacterial populations ([82]). Bet hedging is defined as stochastic switching between phenotypic stages ([83,84]) in order to facilitate persistence in fluctuating environmental conditions. The new host background, representing changed environmental conditions for the symbionts, could have led to the evolution of a bet hedging-like strategy in RC20, switching between two *Wolbachia* variants. Switch from *w*Cer2 to *w*Cer1 is correlated with enhanced fecundity in RC20, and it cannot be ruled out that such adaptive bet hedging results in switching back to *w*Cer2 as main infection variant.

In RC33 we found clear co-existence of *w*Cer1 and *w*Cer2, obviously not subjected to inter-strain competition. Densities of both strains seem to be equal in this system, suggesting competition-free co-existence. This was already shown for the parasitic wasp *Leptopilina heterotoma* where density of different *Wolbachia* strains was not affected by the presence of other strains ([85]). Stable multiple infections with *Wolbachia* were reported from *D. simulans* ([86]), *R. cerasi* ([39]), and *Ae. albopictus* hosts ([87]). The idea of stable co-existence without inter-strain competition of the bacteria is supported by significantly increased rates of maternal transmission determined in this line in comparison to the rate evaluated shortly after microinjection. This is in agreement with our study where *w*Cer2 in *D. simulans* exhibited an initial prevalence of 65% ([29]), and has now reached almost complete transmission (95%; data not shown).

### 4.5 Trans-Infection Events Claim Reproduction Costs In Novel Hosts

[88] demonstrated that *w*MelPop triggers severe phenotypic changes such as decrease of fecundity in the mosquito *Ae. albopictus*. The authors report a clear correlation between host phenotype and the endosymbiont *Wolbachia*. Similar to the situation in mosquitoes, we observed that fecundity of trans-infected *D. simulans* lines is affected by the artificially introduced *Wolbachia* infection when measured ten generations after microinjection ([29]). Data obtained from an ovary screening assay more than 150 generations post microinjection suggested that female flies were still not adapted to the infection. Compared to the uninfected *D. simulans* Nouméa TC strain, 80% of the trans-infected RC lines displayed decreased ovary sizes. Most interestingly, ovaries of RC20 females were significantly larger, as they contained more mature eggs than the other RC lines and slightly larger than the uninfected control. The fecundity of RC20 seemed to change from very poor at the beginning to enhanced in later host generations in the course of this study (DS, personal observation). Hence we might observe a correlation between female fecundity and *Wolbachia* infection in RC20. We have tracked a switch in *w*Cer2/*w*Cer1 prevalence in RC20 that most likely occurred between generations 150 and 167. It might be possible that this change of female fecundity is correlated with a *Wolbachia* strain switch in this line. Although we have no direct evidence yet, we speculate that *w*Cer2 might be negatively correlated with female fecundity in this special case of RC20. This line was reported as mono-infected with *w*Cer2 in earlier passages ([29]), later diagnosed as *w*Cer1&2 double-infected (this study), and eventually the *w*Cer1 infection has outcompeted *w*Cer2, since the latter strain was no longer traceable by PCR in later generations (**Figure 1B**). In order to determine when this shift from *w*Cer2 to *w*Cer1 took place, we analyzed RC20 DNA extracts from seven, randomly picked, non-consecutive generations between G140 (beginning of this study) and G168 (see time course in **Figure 1A**). We found a switch from *w*Cer2 to *w*Cer1 during a transition period between generations F150 and F167, followed by replacement of *w*Cer2 by *w*Cer1 in F168. However, to directly prove the correlation between *w*Cer2 and host fecundity, and to rule out that the microinjection-caused bottleneck did not just lead to accumulation of negative effects, further experiments are needed. Re-evaluation of our data in a homogenized host nuclear background obtained through outcrossing the RC20 line will allow for better analysis of *w*Cer-triggered fitness costs in the host.

## Conclusion

In this study we aimed at testing if artificial symbiont transfer triggers structural rearrangements, and acceleration of SNPs in the symbiont genome. Analysis of mobile genetic elements within *Wolbachia* did not reveal rearrangements after arrival of the symbiont in the recipient hosts. By assessing SNP frequency in three essential *Wolbachia* genes before and after microinjection, we determined that the purifying constraint operating on these loci is hardly relaxed after more than 150 host generations. Instead of tracing new mutations upon transfer in the recipients, we discovered ancestral polymorphic sites in symbiont genes deriving from the donor, pinpointing that both *w*Cer1 and *w*Cer2 exhibit ancestral and cryptic sequence polymorphism in its original host *R. cerasi*. We further uncovered multiple strains in *D. simulans* lines that were previously typed as singly infected. This may have been due the co-existence of *Wolbachia* strains, where one of these persisted at low titer and thus had escaped standard detection techniques. We demonstrated that infections by multiple strains are prone to shifts in strain prevalence upon artificial host transfer. This reflects the population-like structure of *Wolbachia* within and between different hosts and thus will have consequences for symbiont population dynamics. Persistence of cryptic multiple infections after transfer from a multiply infected donor, captures the importance of studying, in detail, the integrity of *Wolbachia* infections prior to application as tools in modern pest and disease control management.

## Supporting Information

Data S1 ****Extended methodology for RFLP-mapping via genomic Southern blot analysis.** Detailed information about RFLP mapping can be found in Data S1.**
(DOCX)

Figure S1 ***Wsp*** **-PCR of antibiotic treated RC20, -50 and** ***R. ceras*** **i.** Upper lane: Generation F1 from 0.03% tetracycline-treated RC20 (1–2) and RC50 (3–4); generation F2 in same order (5–8). RC 50 harbors *Wolbachia* at higher densities than RC20 resulting in still bright signals upon treatment in F1 and highly significant reduction in F2. Positive control (*Wolbachia*-infected DSR) is shown in 9. Lower lane: untreated *R. cerasi* larva (1); antibiotic-treated *R. cerasi* larvae from 0.02%, 0.025%, 0.05% tetracycline (2–4); antibiotic-treated *R. cerasi* adults from 0.02%, 0.025% tetracycline (5–6); untreated *R. cerasi* adult (7). Negative controls (*Wolbachia*-uninfected *D. simulans* Nouméa TC) are shown in 8 and 9. (TIF)

Figure S2 **RFLP analysis of** ***w*** **Cer2 infection in single trans-infected** ***Drosophila*** **(RC)** ***via*** **Southern hybridization.** (**A**) Hybridzation with *IS5*-probe (Insertion Sequence Element), on *Hin*dIII-digested total DNA of *D. melanogaster* Harwich (*w*Mel; *Wol* ^pos^) as reference and trans-infected RC lines RC33 (1), RC45 (2–3), RC50 (4–5), RC21 (6). Black asterisks indicate two fragments that are not present in the *w*Mel reference and are thus considered diagnostic for *w*Cer2. *Hin*dIII-lambda bands 9400, 6600, 4300, 2300, 200. (**B**) Number of fragments in characteristic RFLP-fingerprints of *w*Mel and wCer2 using six different *Wolbachia* marker. **^a^**Expected numbers of fragments after *in silico* analysis of the annotated *w*Mel genome (indicated by asterisk; GenBank accession number NC_002978.6), and **^b^**experimentally determined fragment numbers obtained from RFLP analysis. Number of fixed fragments refers to fragments that are present in the reference *w*Mel and not considered diagnostic for *w*Cer2. (**C**) Hybridization with *IS3* (**D**) *ISNew* (**E**) *VNTR*-141 (**F**) upper panel: *VNTR*-144, lower panel: *wsp*. Abbreviations: M Marker (Lambda-DNA digested with *Hin*dIII). (TIF)

Table S1 **Summary of tested samples deriving from recipient hosts** ***D. simulans*** **and** ***C. capitata*** **.** Numbers in the first column correspond to sequenced clones; second column gives the size of the sequenced gene fragment of either *coxA*, *ftsZ*, or *gatB* of *w*Cer1 and *w*Cer2. Geographic origin and/or collection site for each clone is listed in the last column. (DOCX)

Table S2 **Summary of tested** ***R. cerasi*** **samples.** Numbers in the first column correspond to sequenced clones; second column gives the size of the sequenced gene fragment of either *coxA*, *ftsZ*, or *gatB* of *w*Cer1 and *w*Cer2. Geographic origin and/or collection site for each clone is listed in the last column. (DOCX)

Table S3 **d_S_/d_N_ ratios of** ***gatB*** **.** Table shows d_S_/d_N_ ratios of *gatB* from *w*Cer2 in the donor *R. cerasi* and the recipients *D. simulans* (RC) and *C. capitata* (*Wol*Med88.6). (DOC)

Table S4 **Population-wise sorting of analyzed samples from the** ***w*** **Cer donor** ***Rhagoletis cerasi*** **.** Left column lists names of clones, right column lists the population with the corresponding geographic location. Sequences derive from *gatB* of (**A**) *w*Cer and (**B**) *w*Cer1, plus (**C**) *ftsZ* of *w*Cer1. (DOCX)
