# Supplementary material for: Uncovering Wolbachia Diversity upon Artificial Host Transfer
Source: PLoS One. 2013 Dec 20;8(12):e82402. doi: 10.1371/journal.pone.0082402 (PMC3869692; doi:10.1371/journal.pone.0082402)
Supplement: Data S1 — Extended methodology for RFLP-mapping via genomic Southern blot analysis. Detailed information about RFLP mapping can be found in Data S1. (DOCX) [file pone.0082402.s001.docx]

**Extended Methodology for RFLP Mapping via genomic Southern blot analysis**

**Transposable *IS* Elements**

*w*Cer2 infection was analyzed for structural integrity via Southern hybridization based RFLP-fingerprinting with specific probes targeting the highly variable *Wolbachia* transposons, *IS3* (15 copies in *w*Mel), *IS5* (13 copies in *w*Mel) and *ISNew* (12 copies in *w*Mel; all listed in Table 2 of [40]). Probes were generated according to the IS families discovered in the genome of *w*Mel of *Drosophila melanogaster* (GenBank accession number AE017196). For all three probes, we found no evidence for IS insertion polymorphism or ectopic recombination as RFLP-patterns of *w*Cer2-*Wolbachia* were homogeneous in all 18 samples deriving from trans-infected RC and *Wol*Med88.6 (**Figure S2**). The RFLP-pattern for *w*Cer2 is characterized by 14 *IS3* fragments, six *IS5* fragments (**Figure S2**) and more than eight *ISNew* fragments. Comparison with the RFLP-pattern obtained from *w*Mel of *D. melanogaster* ([40]; and this study), revealed that more than two-thirds of the IS insertions are fixed between this *Wolbachia* infection and *w*Cer2 (Table S2). Rehybridization of the membrane with the maker probes for VNTR-141 and VNTR 144 also gave rise to diagnostic RFLP patterns identical to the ones detected in the donor (data not shown).

**Variable Number Tandem Repeats (VNTRs)**

Structural integrity of *w*Cer2 in recipient hosts was also evaluated utilizing probes targeting two highly variable VNTR loci. Upon *Hin*dIII digestion, independent hybridizations with VNTR-141 probe resulted in RFLP-patterns showing no variability in *w*Cer2 between novel hosts, displayed by six characteristic predominant fragments. We have detected at least 12 more bands of less intensity in the autoradiograph which are most likely due to cross-hybridization with other, non-VNTR-141 loci dispersed in the *w*Cer2 genome (**Figure S2**). *w*Mel was represented by a different RFLP-pattern but at least four fragment positions were found to be fixed between *w*Mel and *w*Cer2 infection (**Figure S2**). We determined one copy of VNTR-144 in *w*Cer2 chromosome represented within a 3.7 kb fragment on the autoradiograph (**Figure S2**). In all tested RC lines (1 x RC20, 1 x RC33, 2 x RC40, 2 x RC50, 1 x RC21), *w*Cer2 infection was represented by the same 3.7 kb fragment, displaying no structural polymorphism. Upon *Hin*dIII digestion, *w*Mel of *D. melanogaster* was also characterized by only one fragment of the same size.

In addition, we performed control hybridizations with a *wsp* probe to demonstrate the presence of *Wolbachia* in tested RC lines plus the *D. melanogaster* reference. *Wsp* is characterized by a single fragment; no signs of a potential double infection represented by a second *wsp* fragment were detected in our sample set (**Figure S2**).
