# Supplementary figures and images for: Uncovering Wolbachia Diversity upon Artificial Host Transfer
Source: PLoS One. 2013 Dec 20;8(12):e82402. doi: 10.1371/journal.pone.0082402 (PMC3869692; doi:10.1371/journal.pone.0082402)

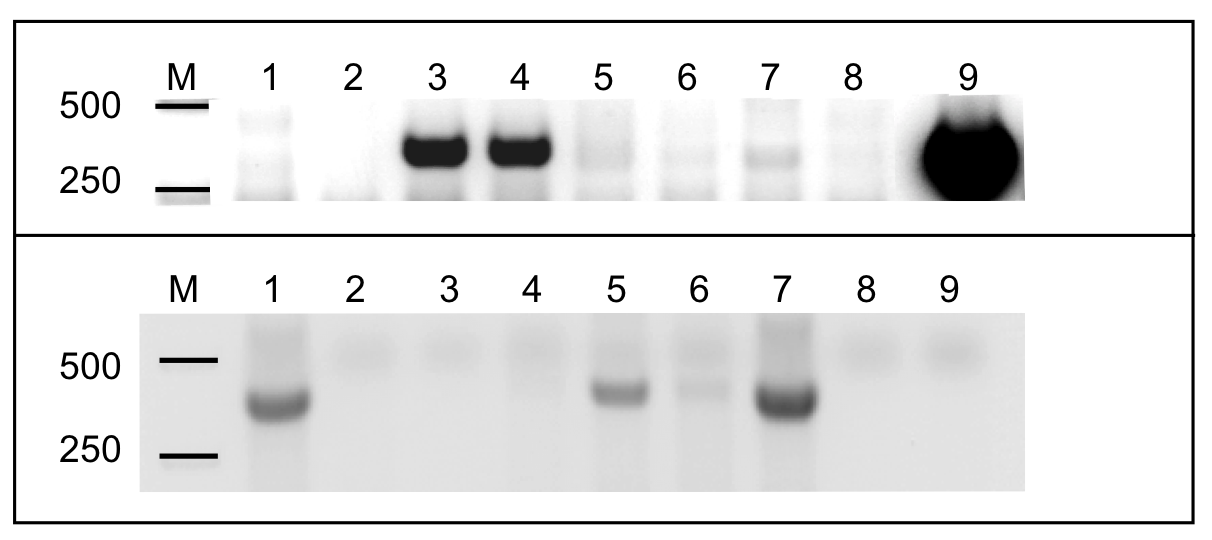

Supplement: Figure S1 — (TIF) [file pone.0082402.s002.tif]

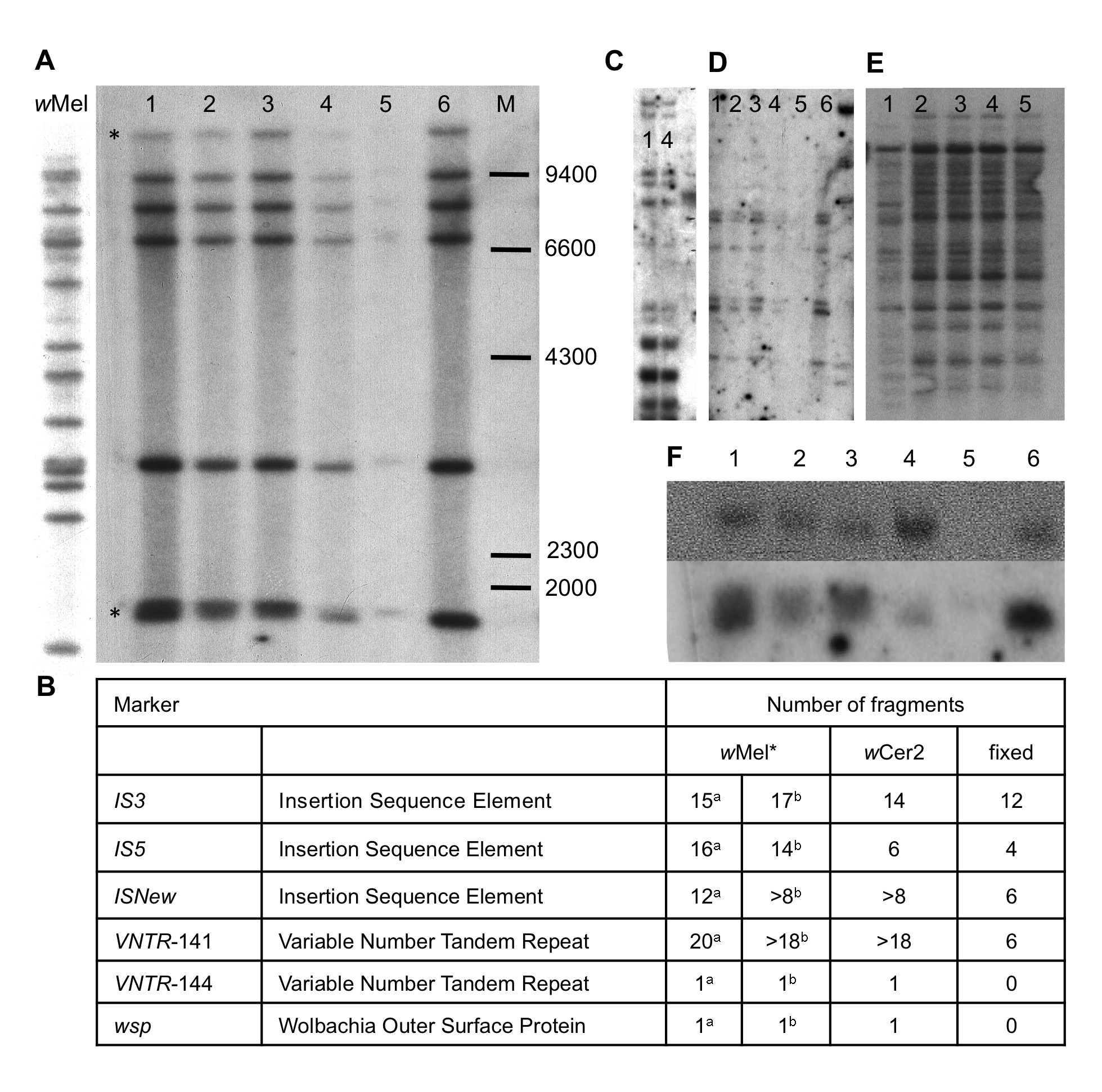

Supplement: Figure S2 — (TIF) [file pone.0082402.s003.tif]
