# Supplementary material for: Uncovering Wolbachia Diversity upon Artificial Host Transfer
Source: PLoS One. 2013 Dec 20;8(12):e82402. doi: 10.1371/journal.pone.0082402 (PMC3869692; doi:10.1371/journal.pone.0082402)
Supplement: Table S1 — (DOCX) [file pone.0082402.s004.docx]

| no. | gene | amplicon size | host system | code | collection site/population |
| --- | --- | --- | --- | --- | --- |
| 1 | *gatB* of *w*Cer1 | 404 bases | *D. simulans* | gatBUDS1_2 | RC20 trans-infected |
| 2 | *gatB* of *w*Cer1 | 404 bases | *D. simulans* | gatBUDS1_3 | RC20 trans-infected |
| 3 | *gatB* of *w*Cer1 | 404 bases | *D. simulans* | gatBUDS1_4 | RC20 trans-infected |
| 1 | *gatB* of *w*Cer2 | 404 bases | *D. simulans* | 257082 | RC20 trans-infected |
| 2 | *gatB* of *w*Cer2 | 404 bases | *D. simulans* | 257084 | RC20 trans-infected |
| 3 | *gatB* of *w*Cer2 | 404 bases | *D. simulans* | 257085 | RC20 trans-infected |
| 4 | *gatB* of *w*Cer2 | 404 bases | *D. simulans* | 257086 | RC20 trans-infected |
| 5 | *gatB* of *w*Cer2 | 404 bases | *D. simulans* | 257089 | RC20 trans-infected |
| 6 | *gatB* of *w*Cer2 | 404 bases | *D. simulans* | 2570811 | RC20 trans-infected |
| 7 | *gatB* of *w*Cer2 | 404 bases | *D. simulans* | 2570812 | RC20 trans-infected |
| 8 | *gatB* of *w*Cer2 | 404 bases | *D. simulans* | 2570814 | RC20 trans-infected |
| 9 | *gatB* of *w*Cer2 | 404 bases | *D. simulans* | 1_1gBU | RC20 trans-infected |
| 10 | *gatB* of *w*Cer2 | 404 bases | *D. simulans* | 3 | RC20 trans-infected |
| 11 | *gatB* of *w*Cer2 | 404 bases | *D. simulans* | 76093 | RC20 trans-infected |
| 12 | *gatB* of *w*Cer2 | 404 bases | *D. simulans* | 76094 | RC20 trans-infected |
| 13 | *gatB* of *w*Cer2 | 404 bases | *D. simulans* | 2570816 | RC33 trans-infected |
| 14 | *gatB* of *w*Cer2 | 404 bases | *D. simulans* | 2570817 | RC33 trans-infected |
| 15 | *gatB* of *w*Cer2 | 404 bases | *D. simulans* | 2570818 | RC33 trans-infected |
| 16 | *gatB* of *w*Cer2 | 404 bases | *D. simulans* | 2570819 | RC33 trans-infected |
| 17 | *gatB* of *w*Cer2 | 404 bases | *D. simulans* | 2570820 | RC33 trans-infected |
| 18 | *gatB* of *w*Cer2 | 404 bases | *D. simulans* | 2570821 | RC33 trans-infected |
| 19 | *gatB* of *w*Cer2 | 404 bases | *D. simulans* | 2570822 | RC33 trans-infected |
| 20 | *gatB* of *w*Cer2 | 404 bases | *D. simulans* | 2570823 | RC33 trans-infected |
| 21 | *gatB* of *w*Cer2 | 404 bases | *D. simulans* | 2_7gBU | RC33 trans-infected |
| 22 | *gatB* of *w*Cer2 | 404 bases | *D. simulans* | 5 | RC33 trans-infected |
| 23 | *gatB* of *w*Cer2 | 404 bases | *D. simulans* | 61 | RC33 trans-infected |
| 24 | *gatB* of *w*Cer2 | 404 bases | *D. simulans* | 76096 | RC33 trans-infected |
| 25 | *gatB* of *w*Cer2 | 404 bases | *D. simulans* | 2570835 | RC45 trans-infected |
| 26 | *gatB* of *w*Cer2 | 404 bases | *D. simulans* | 3_11gBU | RC45 trans-infected |
| 27 | *gatB* of *w*Cer2 | 404 bases | *D. simulans* | 135091 | RC45 trans-infected |
| 28 | *gatB* of *w*Cer2 | 404 bases | *D. simulans* | 135092 | RC45 trans-infected |
| 29 | *gatB* of *w*Cer2 | 404 bases | *D. simulans* | 135093 | RC45 trans-infected |
|  |  |  |  |  |  |
| 1 | *coxA* of *w*Cer1 | 305 bases | *D. simulans* | 2841117 | RC20 trans-infected |
| 2 | *coxA* of *w*Cer1 | 305 bases | *D. simulans* | 2841119 | RC20 trans-infected |
| 3 | *coxA* of *w*Cer1 | 305 bases | *D. simulans* | 2841120 | RC20 trans-infected |
| 4 | *coxA* of *w*Cer1 | 444 bases | *D. simulans* | DS5_43 | RC20 trans-infected |
| 5 | *coxA* of *w*Cer1 | 444 bases | *D. simulans* | DS5_46 | RC20 trans-infected |
| 6 | *coxA* of *w*Cer1 | 444 bases | *D. simulans* | DS5_47 | RC20 trans-infected |
| 7 | *coxA* of *w*Cer1 | 444 bases | *D. simulans* | DS6_51 | RC33 trans-infected |
| 8 | *coxA* of *w*Cer1 | 444 bases | *D. simulans* | DS6_52 | RC33 trans-infected |
| 9 | *coxA* of *w*Cer1 | 444 bases | *D. simulans* | DS6_54 | RC33 trans-infected |
| 10 | *coxA* of *w*Cer1 | 444 bases | *D. simulans* | DS6_57 | RC33 trans-infected |
| 11 | *coxA* of *w*Cer1 | 444 bases | *D. simulans* | DS6_58 | RC33 trans-infected |
| 12 | *coxA* of *w*Cer1 | 444 bases | *C. capitata* | DS79 | *Wol*Med88.6, K. Bourtzis lab, Ioannina, Greece |
|  |  |  |  |  |  |
| 1 | *coxA* of *w*Cer2 | 444 bases | *D. simulans* | DS11 | RC20 trans-infected |
| 2 | *coxA* of *w*Cer2 | 444 bases | *D. simulans* | DS12 | RC20 trans-infected |
| 3 | *coxA* of *w*Cer2 | 444 bases | *D. simulans* | DS5_42 | RC20 trans-infected |
| 4 | *coxA* of *w*Cer2 | 444 bases | *D. simulans* | DS73 | RC33 trans-infected |
| 5 | *coxA* of *w*Cer2 | 444 bases | *D. simulans* | DS74 | RC33 trans-infected |
| 6 | *coxA* of *w*Cer2 | 444 bases | *D. simulans* | DS82 | RC33 trans-infected |
| 7 | *coxA* of *w*Cer2 | 444 bases | *D. simulans* | DS7_3 | RC45 trans-infected |
| 8 | *coxA* of *w*Cer2 | 444 bases | *D. simulans* | DS7_8 | RC45 trans-infected |
| 9 | *coxA* of *w*Cer2 | 444 bases | *D. simulans* | DS7_9 | RC45 trans-infected |
| 10 | *coxA* of *w*Cer2 | 444 bases | *D. simulans* | DS7_10 | RC45 trans-infected |
| 11 | *coxA* of *w*Cer2 | 444 bases | *D. simulans* | DS8_12 | RC45 trans-infected |
| 12 | *coxA* of *w*Cer2 | 444 bases | *D. simulans* | DS8_15 | RC45 trans-infected |
| 13 | *coxA* of *w*Cer2 | 444 bases | *D. simulans* | DS8_18 | RC45 trans-infected |
| 14 | *coxA* of *w*Cer2 | 444 bases | *D. simulans* | DS8_20 | RC45 trans-infected |
| 15 | *coxA* of *w*Cer2 | 444 bases | *D. simulans* | DS9_21 | RC50 trans-infected |
| 16 | *coxA* of *w*Cer2 | 444 bases | *D. simulans* | DS9_24 | RC50 trans-infected |
| 17 | *coxA* of *w*Cer2 | 444 bases | *D. simulans* | DS9_27 | RC50 trans-infected |
| 18 | *coxA* of *w*Cer2 | 444 bases | *D. simulans* | DS9_28 | RC50 trans-infected |
| 19 | *coxA* of *w*Cer2 | 444 bases | *D. simulans* | DS9_29 | RC50 trans-infected |
| 20 | *coxA* of *w*Cer2 | 444 bases | *D. simulans* | DS15 | RC50 trans-infected |
| 21 | *coxA* of *w*Cer2 | 444 bases | *D. simulans* | DS83 | RC50 trans-infected |
| 22 | *coxA* of *w*Cer2 | 444 bases | *D. simulans* | DS84 | RC50 trans-infected |
| 23 | *coxA* of *w*Cer2 | 444 bases | *D. simulans* | DS85 | RC50 trans-infected |
| 24 | *coxA* of *w*Cer2 | 444 bases | *D. simulans* | DS10_34 | RC50 trans-infected |
| 25 | *coxA* of *w*Cer2 | 444 bases | *D. simulans* | DS10_35 | RC50 trans-infected |
| 26 | *coxA* of *w*Cer2 | 444 bases | *D. simulans* | DS10_36 | RC50 trans-infected |
| 27 | *coxA* of *w*Cer2 | 444 bases | *D. simulans* | DS10_37 | RC50 trans-infected |
| 28 | *coxA* of *w*Cer2 | 444 bases | *D. simulans* | DS10_38 | RC50 trans-infected |
| 29 | *coxA* of *w*Cer2 | 444 bases | *D. simulans* | DS11_42 | RC21 trans-infected |
| 30 | *coxA* of *w*Cer2 | 444 bases | *D. simulans* | DS11_44 | RC21 trans-infected |
| 31 | *coxA* of *w*Cer2 | 444 bases | *D. simulans* | DS11_45 | RC21 trans-infected |
| 32 | *coxA* of *w*Cer2 | 444 bases | *D. simulans* | DS11_46 | RC21 trans-infected |
| 33 | *coxA* of *w*Cer2 | 444 bases | *C. capitata* | DS3_22 | *Wol*Med88.6, K. Bourtzis lab, Ioannina, Greece |
| 34 | *coxA* of *w*Cer2 | 444 bases | *C. capitata* | DS3_24 | *Wol*Med88.6, K. Bourtzis lab, Ioannina, Greece |
| 35 | *coxA* of *w*Cer2 | 444 bases | *C. capitata* | DS3_25 | *Wol*Med88.6, K. Bourtzis lab, Ioannina, Greece |
| 36 | *coxA* of *w*Cer2 | 444 bases | *C. capitata* | DS3_27 | *Wol*Med88.6, K. Bourtzis lab, Ioannina, Greece |
| 37 | *coxA* of *w*Cer2 | 444 bases | *C. capitata* | DS3_28 | *Wol*Med88.6, K. Bourtzis lab, Ioannina, Greece |
|  |  |  |  |  |  |
| 1 | *ftsZ* of *w*Cer2 | 478 bases | *D. simulans* | fB_1 | RC33 trans-infected |
| 2 | *ftsZ* of *w*Cer2 | 478 bases | *D. simulans* | fB_2 | RC33 trans-infected |
| 3 | *ftsZ* of *w*Cer2 | 478 bases | *D. simulans* | fB_3 | RC33 trans-infected |
| 4 | *ftsZ* of *w*Cer2 | 478 bases | *D. simulans* | fB_4 | RC33 trans-infected |
| 5 | *ftsZ* of *w*Cer2 | 478 bases | *D. simulans* | fD_1 | RC45 trans-infected |
| 6 | *ftsZ* of *w*Cer2 | 478 bases | *D. simulans* | fD_2 | RC45 trans-infected |
| 7 | *ftsZ* of *w*Cer2 | 478 bases | *D. simulans* | fD_3 | RC45 trans-infected |
| 8 | *ftsZ* of *w*Cer2 | 478 bases | *D. simulans* | fD_4 | RC45 trans-infected |
| 9 | *ftsZ* of *w*Cer2 | 478 bases | *D. simulans* | fE_1 | RC50 trans-infected |
| 10 | *ftsZ* of *w*Cer2 | 478 bases | *D. simulans* | fE_2 | RC50 trans-infected |
| 11 | *ftsZ* of *w*Cer2 | 478 bases | *D. simulans* | fE_3 | RC50 trans-infected |
| 12 | *ftsZ* of *w*Cer2 | 478 bases | *D. simulans* | fE_4 | RC50 trans-infected |
| 13 | *ftsZ* of *w*Cer2 | 478 bases | *D. simulans* | fH_1 | RC21 trans-infected |
| 14 | *ftsZ* of *w*Cer2 | 478 bases | *D. simulans* | DS30 | RC20 trans-infected |
| 15 | *ftsZ* of *w*Cer2 | 478 bases | *D. simulans* | DS31 | RC20 trans-infected |
| 16 | *ftsZ* of *w*Cer2 | 478 bases | *D. simulans* | DS32 | RC20 trans-infected |

**Table S1. Summary of tested samples deriving from trans-infected *D. simulans* and *C. capitata***. Numbers in the first column correspond to sequenced clones; second column gives the size of the sequenced gene fragment of either *gatB, coxA*, or *ftsZ* of *w*Cer1 and *w*Cer2-*Wolbachia*. Geographic origin and/or collection site for each clone is listed in the last column.
