# Supplementary material for: Uncovering Wolbachia Diversity upon Artificial Host Transfer
Source: PLoS One. 2013 Dec 20;8(12):e82402. doi: 10.1371/journal.pone.0082402 (PMC3869692; doi:10.1371/journal.pone.0082402)
Supplement: Table S2 — (DOCX) [file pone.0082402.s005.docx]

| no. | gene | amplicon size | host system | code | collection site/population |
| --- | --- | --- | --- | --- | --- |
| 1 | *gatB* of *w*Cer1 | 404 bases | *R. cerasi* | 1_1 WA | Austria, pop 22(Horitschon, Burgenland) |
| 2 | *gatB* of *w*Cer1 | 404 bases | *R. cerasi* | 1_5 WA | Austria, pop 22(Horitschon, Burgenland) |
| 3 | *gatB* of *w*Cer1 | 404 bases | *R. cerasi* | 1_9 WA | Austria, pop 22(Horitschon, Burgenland) |
| 4 | *gatB* of *w*Cer1 | 404 bases | *R. cerasi* | 2_1 WA | Czech Republic, pop 25 (N49,565 E16,498) |
| 5 | *gatB* of *w*Cer1 | 404 bases | *R. cerasi* | 2_2 WA | Czech Republic, pop 25 (N49,565 E16,498) |
| 6 | *gatB* of *w*Cer1 | 404 bases | *R. cerasi* | 2_3 WA | Czech Republic, pop 25 (N49,565 E16,498) |
| 7 | *gatB* of *w*Cer1 | 404 bases | *R. cerasi* | 2_4 WA | Czech Republic, pop 25 (N49,565 E16,498) |
| 8 | *gatB* of *w*Cer1 | 404 bases | *R. cerasi* | 2_5 WA | Czech Republic, pop 25 (N49,565 E16,498) |
| 9 | *gatB* of *w*Cer1 | 404 bases | *R. cerasi* | 2_6 WA | Czech Republic, pop 25 (N49,565 E16,498) |
| 10 | *gatB* of *w*Cer1 | 404 bases | *R. cerasi* | 2_7 WA | Czech Republic, pop 25 (N49,565 E16,498) |
| 11 | *gatB* of *w*Cer1 | 404 bases | *R. cerasi* | 2_8 WA | Czech Republic, pop 25 (N49,565 E16,498) |
| 12 | *gatB* of *w*Cer1 | 404 bases | *R. cerasi* | 2_9 WA | Czech Republic, pop 25 (N49,565 E16,498) |
| 13 | *gatB* of *w*Cer1 | 404 bases | *R. cerasi* | 2_10 WA | Czech Republic, pop 25 (N49,565 E16,498) |
| 14 | *gatB* of *w*Cer1 | 404 bases | *R. cerasi* | 2_11 WA | Czech Republic, pop 25 (N49,565 E16,498) |
| 15 | *gatB* of *w*Cer1 | 404 bases | *R. cerasi* | 2_12 WA | Czech Republic, pop 25 (N49,565 E16,498) |
| 16 | *gatB* of *w*Cer1 | 404 bases | *R. cerasi* | DS11_1 | Czech Republic, pop 28 (N49,085 E16,589) |
| 17 | *gatB* of *w*Cer1 | 404 bases | *R. cerasi* | DS1 | Hungary |
| 18 | *gatB* of *w*Cer1 | 404 bases | *R. cerasi* | DS2 | Hungary |
| 19 | *gatB* of *w*Cer1 | 404 bases | *R. cerasi* | DS35 | Romania |
| 20 | *gatB* of *w*Cer1 | 404 bases | *R. cerasi* | DS36 | Romania |
| 21 | *gatB* of *w*Cer1 | 404 bases | *R. cerasi* | DS57 | Austria, (Hietzing, Vienna) |
| 22 | *gatB* of *w*Cer1 | 404 bases | *R. cerasi* | DS58 | Austria, (Hietzing, Vienna) |
| 23 | *gatB* of *w*Cer1 | 404 bases | *R. cerasi* | DS62 | Austria, (Hietzing, Vienna) |
| 24 | *gatB* of *w*Cer1 | 404 bases | *R. cerasi* | DS63 | Austria, (Hietzing, Vienna) |
| 1 | *gatB* of *w*Cer2 | 404 bases | *R. cerasi* | 284094 | Italy, pop 7 (Bivona, Sicily) |
| 2 | *gatB* of *w*Cer2 | 404 bases | *R. cerasi* | 284099 | Austria, pop 20 (Loosdorf, Burgenland) |
| 3 | *gatB* of *w*Cer2 | 404 bases | *R. cerasi* | 2840913 | Austria, pop 20 (Loosdorf, Burgenland) |
| 4 | *gatB* of *w*Cer2 | 404 bases | *R. cerasi* | 2840920 | Italy, pop 41 (Chiusa Sclafani, Sicily) |
| 5 | *gatB* of *w*Cer2 | 404 bases | *R. cerasi* | 2840934 | Italy, pop 7 (Bivona, Sicily) |
| 6 | *gatB* of *w*Cer2 | 404 bases | *R. cerasi* | 2840939 | Austria, pop 20 (Loosdorf, Burgenland) |
| 7 | *gatB* of *w*Cer2 | 404 bases | *R. cerasi* | 1_7 WARC_Aut22 | Austria, pop 22(Horitschon, Burgenland) |
| 8 | *gatB* of *w*Cer2 | 404 bases | *R. cerasi* | 1_12 WARC_Aut22 | Austria, pop 22 (Horitschon, Burgenland) |
| 9 | *gatB* of *w*Cer2 | 404 bases | *R. cerasi* | 1150939 | Italy, pop 7 (Bivona, Sicily) |
| 10 | *gatB* of *w*Cer2 | 404 bases | *R. cerasi* | 1150940 | Italy, pop 7 (Bivona, Sicily) |
| 11 | *gatB* of *w*Cer2 | 404 bases | *R. cerasi* | 1150941 | Italy, pop 7 (Bivona, Sicily) |
| 12 | *gatB* of *w*Cer2 | 404 bases | *R. cerasi* | 1150942 | Italy, pop 7 (Bivona, Sicily) |
| 13 | *gatB* of *w*Cer2 | 404 bases | *R. cerasi* | 1150943 | Italy, pop 7 (Bivona, Sicily) |
| 14 | *gatB* of *w*Cer2 | 404 bases | *R. cerasi* | 1150945 | Italy, pop 7 (Bivona, Sicily) |
| 15 | *gatB* of *w*Cer2 | 404 bases | *R. cerasi* | 1150946 | Italy, pop 7 (Bivona, Sicily) |
| 16 | *gatB* of *w*Cer2 | 404 bases | *R. cerasi* | 1150947 | Italy, pop 7 (Bivona, Sicily) |
| 17 | *gatB* of *w*Cer2 | 404 bases | *R. cerasi* | 1150951 | Austria, pop 22 (Horitschon, Burgenland) |
| 18 | *gatB* of *w*Cer2 | 404 bases | *R. cerasi* | 1150952 | Austria, pop 22 (Horitschon, Burgenland) |
| 19 | *gatB* of *w*Cer2 | 404 bases | *R. cerasi* | 1150953 | Austria, pop 22 (Horitschon, Burgenland) |
| 20 | *gatB* of *w*Cer2 | 404 bases | *R. cerasi* | 1150954 | Austria, pop 22 (Horitschon, Burgenland) |
| 21 | *gatB* of *w*Cer2 | 404 bases | *R. cerasi* | 1250918 | Italy, pop 7 (Bivona, Sicily) |
| 22 | *gatB* of *w*Cer2 | 404 bases | *R. cerasi* | 1250919 | Italy, pop 7 (Bivona, Sicily) |
|  |  |  |  |  |  |
| 1 | *coxA* of *w*Cer1 | 444 bases | *R. cerasi* | DS1_3 | Italy, pop10 (Lingua Glossa, Sicily) |
| 2 | *coxA* of *w*Cer1 | 444 bases | *R. cerasi* | DS1_4 | Italy, pop10 (Lingua Glossa, Sicily) |
| 3 | *coxA* of *w*Cer1 | 444 bases | *R. cerasi* | DS1_7 | Italy, pop10 (Lingua Glossa, Sicily) |
| 4 | *coxA* of *w*Cer1 | 444 bases | *R. cerasi* | DS1_8 | Italy, pop10 (Lingua Glossa, Sicily) |
| 5 | *coxA* of *w*Cer1 | 444 bases | *R. cerasi* | DS1_9 | Italy, pop10 (Lingua Glossa, Sicily) |
| 6 | *coxA* of *w*Cer1 | 444 bases | *R. cerasi* | DS1_10 | Italy, pop10 (Lingua Glossa, Sicily) |
| 7 | *coxA* of *w*Cer1 | 444 bases | *R. cerasi* | DS2_11 | Czech Republic, pop 28 (N49,085 E16,589) |
| 8 | *coxA* of *w*Cer1 | 444 bases | *R. cerasi* | DS2_13 | Czech Republic, pop 28 (N49,085 E16,589) |
| 9 | *coxA* of *w*Cer1 | 444 bases | *R. cerasi* | DS2_17 | Czech Republic, pop 28 (N49,085 E16,589) |
| 10 | *coxA* of *w*Cer1 | 444 bases | *R. cerasi* | DS2_19 | Czech Republic, pop 28 (N49,085 E16,589) |
| 11 | *coxA* of *w*Cer1 | 444 bases | *R. cerasi* | DS2_20 | Czech Republic, pop 28 (N49,085 E16,589) |
| 12 | *coxA* of *w*Cer1 | 444 bases | *R. cerasi* | DS70 | Romania |
| 13 | *coxA* of *w*Cer1 | 444 bases | *R. cerasi* | DS71 | Romania |
| 1 | *coxA* of *w*Cer2 | 444 bases | *R. cerasi* | DS69 | Austria, (Hietzing, Vienna) |
| 2 | *coxA* of *w*Cer2 | 444 bases | *R. cerasi* | DS77 | Austria, (Hietzing, Vienna) |
|  |  |  |  |  |  |
| 1 | *ftsZ* of *w*Cer1 | 478 bases | *R. cerasi* | DS27 | Hungary |
| 2 | *ftsZ* of *w*Cer1 | 478 bases | *R. cerasi* | DS29 | Hungary |
| 3 | *ftsZ* of *w*Cer1 | 478 bases | *R. cerasi* | f1_1 | Czech Republic, pop 25 (N49,565 E16,498) |
| 4 | *ftsZ* of *w*Cer1 | 478 bases | *R. cerasi* | f1_3 | Czech Republic, pop 25 (N49,565 E16,498) |
| 5 | *ftsZ* of *w*Cer1 | 478 bases | *R. cerasi* | f1_4 | Czech Republic, pop 25 (N49,565 E16,498) |
| 6 | *ftsZ* of *w*Cer1 | 478 bases | *R. cerasi* | f2_1 | Czech Republic, pop 27 (N49,372 E16,553) |
| 7 | *ftsZ* of *w*Cer1 | 478 bases | *R. cerasi* | f2_2 | Czech Republic, pop 27 (N49,372 E16,553) |
| 8 | *ftsZ* of *w*Cer1 | 478 bases | *R. cerasi* | f2_3 | Czech Republic, pop 27 (N49,372 E16,553) |
| 9 | *ftsZ* of *w*Cer1 | 478 bases | *R. cerasi* | f2_4 | Czech Republic, pop 27 (N49,372 E16,553) |
| 10 | *ftsZ* of *w*Cer1 | 478 bases | *R. cerasi* | f3_2 | Czech Republic, pop 28 (N49,085 E16,589) |
| 11 | *ftsZ* of *w*Cer1 | 478 bases | *R. cerasi* | f3_3 | Czech Republic, pop 28 (N49,085 E16,589) |
| 12 | *ftsZ* of *w*Cer1 | 478 bases | *R. cerasi* | f3_4 | Czech Republic, pop 28 (N49,085 E16,589) |
| 13 | *ftsZ* of *w*Cer1 | 478 bases | *R. cerasi* | f4_4 | Austria, pop 4 (Neufeld, Burgenland) |
| 14 | *ftsZ* of *w*Cer1 | 478 bases | *R. cerasi* | f37_1_1 | Italy, pop 37 (Passopomo, Sicily) |
| 15 | *ftsZ* of *w*Cer1 | 478 bases | *R. cerasi* | f38_1_1 | Italy, pop 38 (Via Grande, Sicily) |
| 16 | *ftsZ* of *w*Cer1 | 478 bases | *R. cerasi* | f38_1_2 | Italy, pop 38 (Via Grande, Sicily) |
| 17 | *ftsZ* of *w*Cer1 | 478 bases | *R. cerasi* | f38_1_4 | Italy, pop 38 (Via Grande, Sicily) |
| 18 | *ftsZ* of *w*Cer1 | 478 bases | *R. cerasi* | f39_1_2 | Italy, pop 39(Chiusa Sclafani, Sicily) |
| 19 | *ftsZ* of *w*Cer1 | 478 bases | *R. cerasi* | f40_2_3 | Italy, pop 40 (Chiusa Sclafani, Sicily) |
| 20 | *ftsZ* of *w*Cer1 | 478 bases | *R. cerasi* | f40_2_4 | Italy, pop 40 (Chiusa Sclafani, Sicily) |
| 21 | *ftsZ* of *w*Cer1 | 478 bases | *R. cerasi* | f41_2_3 | Italy, pop 41 (Chiusa Sclafani, Sicily) |
| 22 | *ftsZ* of *w*Cer1 | 478 bases | *R. cerasi* | f41_2_4 | Italy, pop 41 (Chiusa Sclafani, Sicily) |
| 1 | *ftsZ* of *w*Cer2 | 478 bases | *R. cerasi* | DS28 | Hungary |
| 2 | *ftsZ* of *w*Cer2 | 478 bases | *R. cerasi* | f38_1_2 | Italy, pop 38 (Via Grande, Sicily) |
| 3 | *ftsZ* of *w*Cer2 | 478 bases | *R. cerasi* | f39_1_1 | Italy, pop 39(Chiusa Sclafani, Sicily) |
| 4 | *ftsZ* of *w*Cer2 | 478 bases | *R. cerasi* | f4_1 | Austria, pop 4 (Neufeld, Burgenland) |
| 5 | *ftsZ* of *w*Cer2 | 478 bases | *R. cerasi* | f4_3 | Austria, pop 4 (Neufeld, Burgenland) |
| 6 | *ftsZ* of *w*Cer2 | 478 bases | *R. cerasi* | f42_2_1 | Italy, pop 42 (San Giuseppe, Sicily) |
| 7 | *ftsZ* of *w*Cer2 | 478 bases | *R. cerasi* | f42_2_2 | Italy, pop 42 (San Giuseppe, Sicily) |
| 8 | *ftsZ* of *w*Cer2 | 478 bases | *R. cerasi* | f42_2_3 | Italy, pop 42 (San Giuseppe, Sicily) |
| 9 | *ftsZ* of *w*Cer2 | 478 bases | *R. cerasi* | f42_2_4 | Italy, pop 42 (San Giuseppe, Sicily) |
| 10 | *ftsZ* of *w*Cer2 | 478 bases | *R. cerasi* | f5_1 |  |
| 11 | *ftsZ* of *w*Cer2 | 478 bases | *R. cerasi* | f5_2 |  |
| 12 | *ftsZ* of *w*Cer2 | 478 bases | *R. cerasi* | f5_3 |  |
| 13 | *ftsZ* of *w*Cer2 | 478 bases | *R. cerasi* | f5_4 |  |
| 14 | *ftsZ* of *w*Cer2 | 478 bases | *R. cerasi* | f6_1 |  |
| 15 | *ftsZ* of *w*Cer2 | 478 bases | *R. cerasi* | f6_2 |  |
| 16 | *ftsZ* of *w*Cer2 | 478 bases | *R. cerasi* | f6_3 |  |
| 17 | *ftsZ* of *w*Cer2 | 478 bases | *R. cerasi* | f6_4 |  |
| 18 | *ftsZ* of *w*Cer2 | 478 bases | *R. cerasi* | f7_1 |  |
| 19 | *ftsZ* of *w*Cer2 | 478 bases | *R. cerasi* | f7_2 |  |
| 20 | *ftsZ* of *w*Cer2 | 478 bases | *R. cerasi* | f7_3 |  |
| 21 | *ftsZ* of *w*Cer2 | 478 bases | *R. cerasi* | f7_4 |  |

**Table S2. Summary of tested *R. cerasi* samples**. Numbers in the first column correspond to sequenced clones; second column gives the size of the sequenced gene fragment of either *gatB*, *coxA*, or *ftsZ* of *w*Cer1 and *w*Cer2-*Wolbachia*. Geographic origin and/or collection site for each clone is listed in the last column.
