# Supplementary material for: Uncovering Wolbachia Diversity upon Artificial Host Transfer
Source: PLoS One. 2013 Dec 20;8(12):e82402. doi: 10.1371/journal.pone.0082402 (PMC3869692; doi:10.1371/journal.pone.0082402)
Supplement: Table S3 — (DOC) [file pone.0082402.s006.doc]

|  |  |  |  |  |  |  |  |  |
| --- | --- | --- | --- | --- | --- | --- | --- | --- |
|  |  | **d_S_^1^** | **d_N_^2^** | **d_N_/d_S_ (ω)** | **SE** | ***P* values^3^** | | |
| 1 | *R. cerasi* | 0.0119 | 0.0036 | 3.4824 | ± 0.216759 |  |  |  |
| 2 | RC20 | 0.0252 | 0.0047 | 6.5356 | ± 0.733363 | 2 *vs* 1 | 0.0302 | * |
| 3 | RC33 | 0.0119 | 0.0042 | 3.1748 | ± 0.640391 | 3 *vs* 1 | 0.5694 | N.S. |
| 4 | RC45 | 0.0149 | 0.0031 | 4.7724 | ± 0.632905 | 4 *vs* 1 | 0.0379 | * |
| 5 | RC50 | 0.0118 | 0.0036 | 3.4587 | ± 0.213514 | 5 *vs* 1 | 0.9385 | N.S. |
| 6 | *Wol*Med88.6 | 0.0118 | 0.0106 | 1.1423 | ± 0.054614 | 6 *vs* 1 | <0.0001 | *** |

**Table S3.** d_S_/d_N_ ratios of *gatB* from *w*Cer2 in donor *R. cerasi* and recipients RC of *D. simulans* and *Wol*Med88.6 of *C. capitata*. ^1^d**_S_** = synonymous substitutions, average value; ^2^d**_N_** = non-synonymous substitutions, average value. ^3^Two-tailed *P* values from unpaired *t* tests, values are significant when P<0.05. Abbreviations: SE standard error of the mean.
