# Supplementary material for: Uncovering Wolbachia Diversity upon Artificial Host Transfer
Source: PLoS One. 2013 Dec 20;8(12):e82402. doi: 10.1371/journal.pone.0082402 (PMC3869692; doi:10.1371/journal.pone.0082402)
Supplement: Table S4 — (DOCX) [file pone.0082402.s007.docx]

| **A** | |
| --- | --- |
| *w*Cer2 | *gatB* |
| 284099 | Austria, pop 20 (Loosdorf, Burgenland) |
| 2840913 | Austria, pop 20 (Loosdorf, Burgenland) |
| 2840939 | Austria, pop 20 (Loosdorf, Burgenland) |
| 1_7 WARC_Aut22 | Austria, pop 22(Horitschon, Burgenland) |
| 1_12 WARC_Aut22 | Austria, pop 22 (Horitschon, Burgenland) |
| 1150951 | Austria, pop 22 (Horitschon, Burgenland) |
| 1150952 | Austria, pop 22 (Horitschon, Burgenland) |
| 1150953 | Austria, pop 22 (Horitschon, Burgenland) |
| 1150954 | Austria, pop 22 (Horitschon, Burgenland) |
|  |  |
| **B** |  |
| *w*Cer1 | *gatB* |
| 1_1 WA | Austria, pop 22(Horitschon, Burgenland) |
| 1_5 WA | Austria, pop 22(Horitschon, Burgenland) |
| 1_9 WA | Austria, pop 22(Horitschon, Burgenland) |
| 2_1 WA | Czech Republic, pop 25 (N49,565 E16,498) |
| 2_2 WA | Czech Republic, pop 25 (N49,565 E16,498) |
| 2_3 WA | Czech Republic, pop 25 (N49,565 E16,498) |
| 2_4 WA | Czech Republic, pop 25 (N49,565 E16,498) |
| 2_5 WA | Czech Republic, pop 25 (N49,565 E16,498) |
| 2_6 WA | Czech Republic, pop 25 (N49,565 E16,498) |
| 2_7 WA | Czech Republic, pop 25 (N49,565 E16,498) |
| 2_8 WA | Czech Republic, pop 25 (N49,565 E16,498) |
| 2_9 WA | Czech Republic, pop 25 (N49,565 E16,498) |
| 2_10 WA | Czech Republic, pop 25 (N49,565 E16,498) |
| 2_11 WA | Czech Republic, pop 25 (N49,565 E16,498) |
| 2_12 WA | Czech Republic, pop 25 (N49,565 E16,498) |
| DS11_1 | Czech Republic, pop 28 (N49,085 E16,589) |
|  |  |
| **C** |  |
| *w*Cer1 | *ftsZ* |
| f1_1 | Czech Republic, pop 25 (N49,565 E16,498) |
| f1_3 | Czech Republic, pop 25 (N49,565 E16,498) |
| f1_4 | Czech Republic, pop 25 (N49,565 E16,498) |
| f2_1 | Czech Republic, pop 27 (N49,372 E16,553) |
| f2_2 | Czech Republic, pop 27 (N49,372 E16,553) |
| f2_3 | Czech Republic, pop 27 (N49,372 E16,553) |
| f2_4 | Czech Republic, pop 27 (N49,372 E16,553) |
| f3_2 | Czech Republic, pop 28 (N49,085 E16,589) |
| f3_3 | Czech Republic, pop 28 (N49,085 E16,589) |
| f3_4 | Czech Republic, pop 28 (N49,085 E16,589) |
| f4_4 | Austria, pop 4 (Neufeld, Burgenland) |

Table S4. Population-wise sorting of analyzed samples from the *w*Cer donor *Rhagoletis cerasi*. Left column lists names of clones, right column lists the population with the corresponding geographic location. Sequences derive from *gatB* of **(A)** *w*Cer and **(B)** *w*Cer1, plus **(C)** *ftsZ* of *w*Cer1.
